# Suppression of SIRT1 in Diabetic Conditions Induces Osteogenic Differentiation of Human Vascular Smooth Muscle Cells via RUNX2 Signalling

**DOI:** 10.1038/s41598-018-37027-2

**Published:** 2019-01-29

**Authors:** F. Bartoli-Leonard, F. L. Wilkinson, A. Schiro, F. Serracino Inglott, M. Y. Alexander, R. Weston

**Affiliations:** 1 Translational Cardiovascular Science, Centre for Bioscience, Manchester Metropolitan University, Manchester, UK; 2 Vascular Unit, Manchester NHS Foundation Trust, Manchester Academic Health Science Centre, Manchester, UK

## Abstract

Vascular calcification is associated with significant morbidity and mortality within diabetes, involving activation of osteogenic regulators and transcription factors. Recent evidence demonstrates the beneficial role of Sirtuin 1 (SIRT1), an NAD^+^ dependant deacetylase, in improved insulin sensitivity and glucose homeostasis, linking hyperglycaemia and SIRT1 downregulation. This study aimed to determine the role of SIRT1 in vascular smooth muscle cell (vSMC) calcification within the diabetic environment. An 80% reduction in SIRT1 levels was observed in patients with diabetes, both in serum and the arterial smooth muscle layer, whilst both RUNX2 and Osteocalcin levels were elevated. Human vSMCs exposed to hyperglycaemic conditions *in vitro* demonstrated enhanced calcification, which was positively associated with the induction of cellular senescence, verified by senescence-associated β-galactosidase activity and cell cycle markers p16 and p21. Activation of SIRT1 by SRT1720 reduced Alizarin red staining by a third, via inhibition of the RUNX2 pathway and prevention of senescence. Conversely, inhibition of SIRT1 via Sirtinol and siRNA increased RUNX2 by over 50%. These findings demonstrate the key role that SIRT1 plays in preventing calcification in a diabetic environment, through the inhibition of RUNX2 and senescence pathways, suggesting a downregulation of SIRT1 may be responsible for perpetuating vascular calcification in diabetes.

## Introduction

Diabetes mellitus (DM) is a leading cause of cardiovascular mortality, and with over 422 million cases worldwide, it is ranked as one of the top four diseases to target for development of novel therapies by the World Health Organisation^1^. DM is a major independent risk factor for coronary artery disease, accelerating the development of atherosclerosis and vascular dysfunction^2^, with diabetic complications being a leading cause of patient mortality^3^. Chronic hyperglycaemia, a common pathology of DM, often leads to widespread calcification^4^, which is currently untreatable. Despite blood pressure control and lipid modification therapy to correct hyperglycaemia and atherogenic dyslipidaemia, calcification in the vasculature and associated complications are highly prevalent in the diabetic patient^5^, increasing critical limb ischaemia^6^ and cardiovascular disease risk by 3-fold and 4-fold respectively^7^. Calcification in the diabetic patient is recognised as a strong predictor of lower limb amputation and subsequent cardiovascular mortality^8^.

Vascular calcification (VC) is highly correlated with increased cardiovascular disease (CVD) risk, particularly in patients within DM, which dramatically accelerates the development of atherosclerosis, leading to a hardening of the arteries^9^, a loss of vascular compliance and the development of plaque. It is now accepted that calcification is a cell-mediated process, resembling osteogenesis via vascular smooth muscle cell (vSMC) trans-differentiation into osteoblast-like cells, rather than a passive mineral precipitation as previously thought^10,11^. The aetiology of this pathological process under different conditions has been reviewed extensively^12–14^ and subsequently acknowledged that the deposition of hydroxyapatite occurs at the final stage of the process^15^; however, the composition of hydroxyapatite crystals and the factors triggering VC differs, depending on the disease conditions^16–18^.

Evidence shows that VC involves a loss of mineralisation inhibitory molecules, an induction of osteogenic differentiation factors and increased cellular senescence and apoptosis^19^. Current cellular models have demonstrated that an increase in phosphate and calcium levels, as well as hyperglycaemia play a pivotal role in VC development^20^, however, strategies to control calcium and inorganic phosphate levels in patients have been met with mixed success and there is little to no clinical management in the prevention of calcified matrix deposition.

Sirtuin proteins are a family of seven highly conserved nicotinamide adenine dinucleotide (NAD) + dependent class III histone deacetylases in mammalian cells^21^, whose activity has been associated with cellular metabolism, protection against DNA damage and longevity^22^. SIRT1 activation is induced by increased ionised NAD, and conversely a shift in the NADH/NAD + ratio, commonly observed in hyperglycaemia, decreases SIRT1 expression, potentially leading to detrimental effects in the cell^22^. SIRT1 has been shown to attenuate hyperphosphatemia-induced arterial calcification, by preventing osteoblastic differentiation of human aortic SMCs *in vitro*^23^, although the mechanism is still unclear. In addition, resveratrol, an activator of SIRT1, appears to have a protective effect against vascular calcification in a rat smooth muscle cell model of calcification^24^. The novel SIRT1 activator, SRT1720, is well tolerated within diabetic mice, whilst predominantly having neutral effects on markers of endothelial function and platelet-monocyte function^25^, it can reduce the production of pro-inflammatory cytokines and thus, inflammasome activation. Given the links between inflammation and calcification^26,27^, SIRT1 activation has considerable appeal as an attractive target for an anti-calcification strategy within a diabetic setting. This study now extends these reports and confirms the suppression of SIRT1 in diabetic patients both systemically and within the vascular wall and identifies the mechanism underpinning the protective effects of SIRT1 against vSMC cell matrix deposition in hyperglycaemic conditions *in vitro*, involving senescence and RUNX2 signalling.

## Results

### SIRT1 protein is supressed in diabetic patients

**Figure 1:**
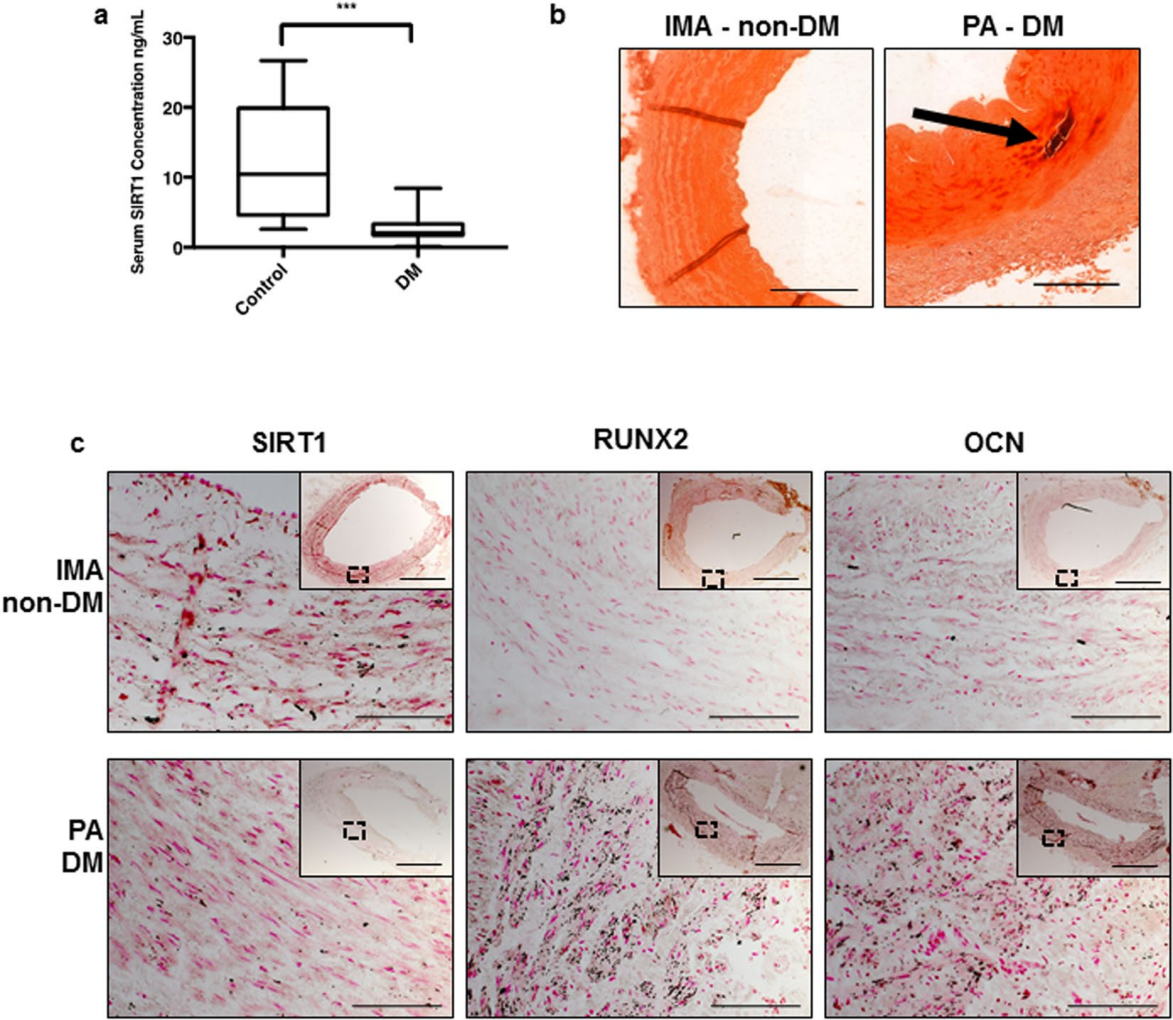
SIRT1 protein is reduced in diabetic patients. The concentration of SIRT1 within patient serum was measured by ELISA. (**a**) SIRT1 serum concentration was significantly decreased by over 75% in diabetic patients (n = 25) compared to healthy controls (n = 25). Representative micrographs of internal mammary artery (IMA) from a non-diabetic patient undergoing CABG surgery, and popliteal artery (PA) harvested from a diabetic patient (DM) undergoing lower limb amputation. (**b**) Alizarin red staining, positive staining shown by arrow (n = 3). (**c**) LH panel showing SIRT1 staining (black), lacking in the DM patient (n = 3). Centre panel showing RUNX2 staining (n = 3) and RH panel showing OCN staining (n = 3), positive stain observed in the DM compared to the IMA. **P < 0.005 Scale bar = 200 μm/20 μm.

Previous studies have shown that serum from patients with DM accelerates osteogenic differentiation and mineralization of vSMCs *in vitro*^28,29^. Since SIRT1 activation by resveratrol has been shown to prevent VC in a rat smooth muscle cell calcification model^24^, and the nuclear sirtuins (SIRT 1, 2, 6 and 7) have been shown to be key players in the regulation of inflammatory responses^30^, this study sought to establish whether loss of SIRT1 could be involved in diabetic-induced VC. Levels of serum SIRT1 were found to be six-fold lower in patients with diabetes (2.076 ng/mL SEM±1.036) compared to healthy controls (15.76 ng/mL SEM±2.184) (Fig. 1a). Alizarin red staining was used to confirm positive calcification in diabetic tissue, which was absent in the non-diabetic internal mammary artery (IMA) (Fig. 1b). Of note, SIRT1 positive staining was evident in IMA; a non-atherosclerotic tissue from a non-diabetic patient, which is typically refractory to atherosclerotic plaque development and mineral deposition^31^. Whilst SIRT1 staining was clearly suppressed in popliteal arterial tissue from diabetic patients with critical limb ischemia (Fig. 1c). The lack of SIRT1 staining and the presence of calcification was observed in all diabetic tissue sections examined within the study. In addition, both the osteogenic markers, RUNX2 and OCN protein localisation was strongly apparent within diabetic patient vessels, which was not observed in the IMA controls (Fig. 1c) (Supplementary Fig. S1).

### Hyperglycaemic conditions increase smooth muscle cell osteogenic differentiation *in vitro*

**Figure 2:**
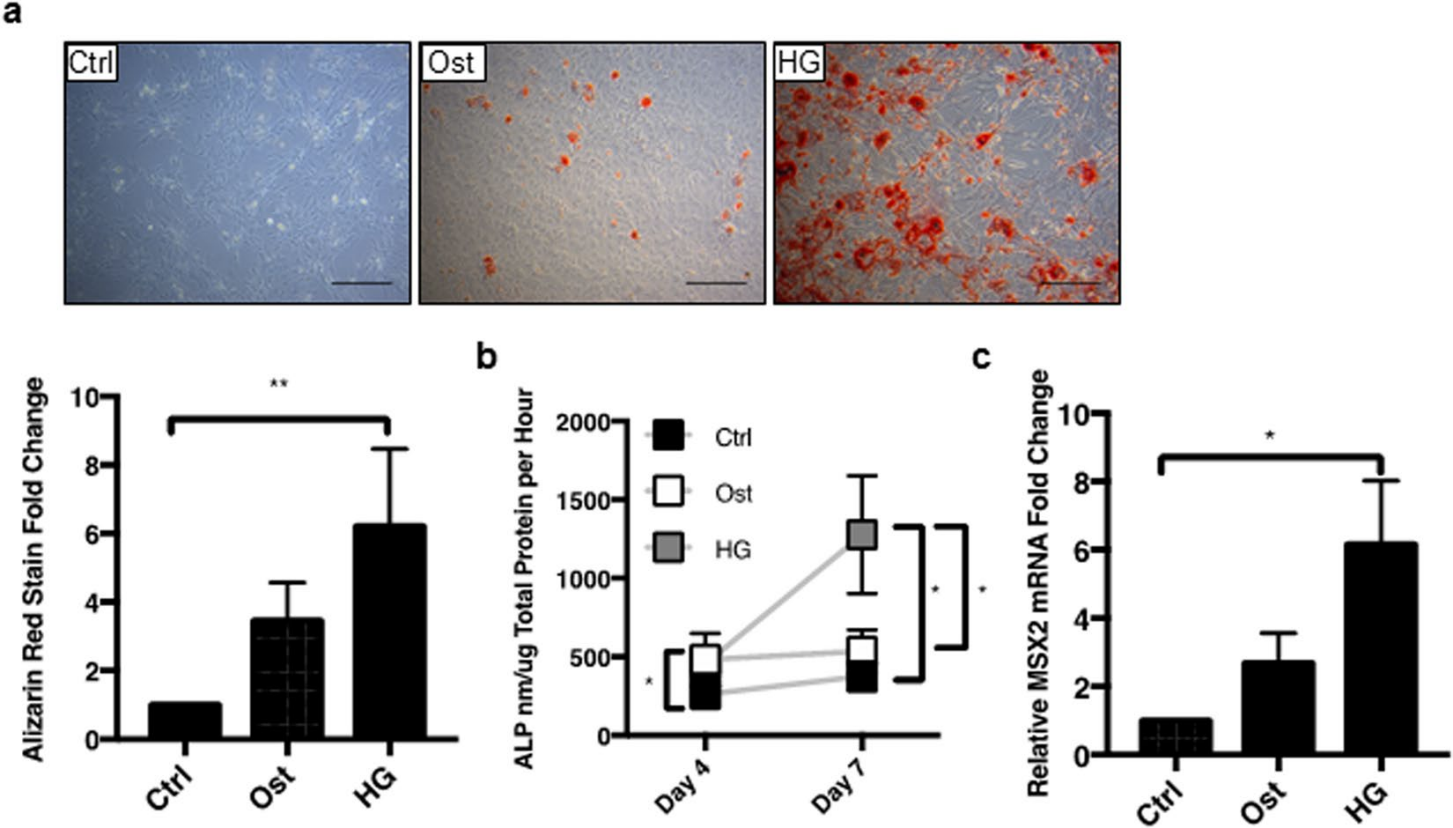
Hyperglycaemic conditions induce smooth muscle cell mineralisation. The effect of control (Ctrl), CaCl_2_ and BGP osteogenic (Ost) and osteogenic with high glucose (HG) conditions was assessed. (**a**) Representative micrographs (n = 4 and 5FoV) show positive Alizarin red staining in osteogenic conditions by day 21, which is significantly greater in high glucose osteogenic media. (**b**) ALP activity was increased by day 4 in hyperglycaemic conditions and increased by over a two-fold in hyperglycaemic conditions compared to osteogenic conditions by day 7 (n = 5). (**c**) MSX2 mRNA expression significantly increased in hyperglycaemic conditions compared to control (n = 3). *P < 0.05, **P < 0.005, ***P < 0.001. Scale Bars = 100 μm

To determine whether physiologically relevant hyperglycaemic conditions effect SIRT1 expression and mineralisation of vSMCs, a well-established *in vitro* calcification model was used. Cells were grown in the presence of elevated CaCl_2_ and βGP (osteogenic; Ost) and in the additional presence or absence of high levels of glucose (hyperglycaemic, HG). As expected, cells cultured under osteogenic conditions deposited a mineralised matrix at day 21, shown by Alizarin Red staining, an effect not detected under control untreated conditions. Of note, the hyperglycaemic media significantly enhanced the osteogenic capacity of the vSMCs, compared to both control and osteogenic conditions (p < 0.0037) (Fig. 2a). Additional confirmation of hyperglycaemic-induced calcification *in vitro* was established by assessment of ALP activity; an important component of hard tissue formation^32^, which was increased in cells in osteogenic media at both day 4 and day 7, compared to the untreated controls (p < 0.0103) (Fig. 2b), and with a further significant increase in ALP activity following culture in hyperglycaemic media (p < 0.0238). Further validation of osteogenesis was confirmed by investigation of MSX2, a known transcriptional regulator of bone development^33^. A significant upregulation of MSX2 was apparent in hyperglycaemic conditions compared to control (p < 0.0468) (Fig. 2c).

**Figure 3:**
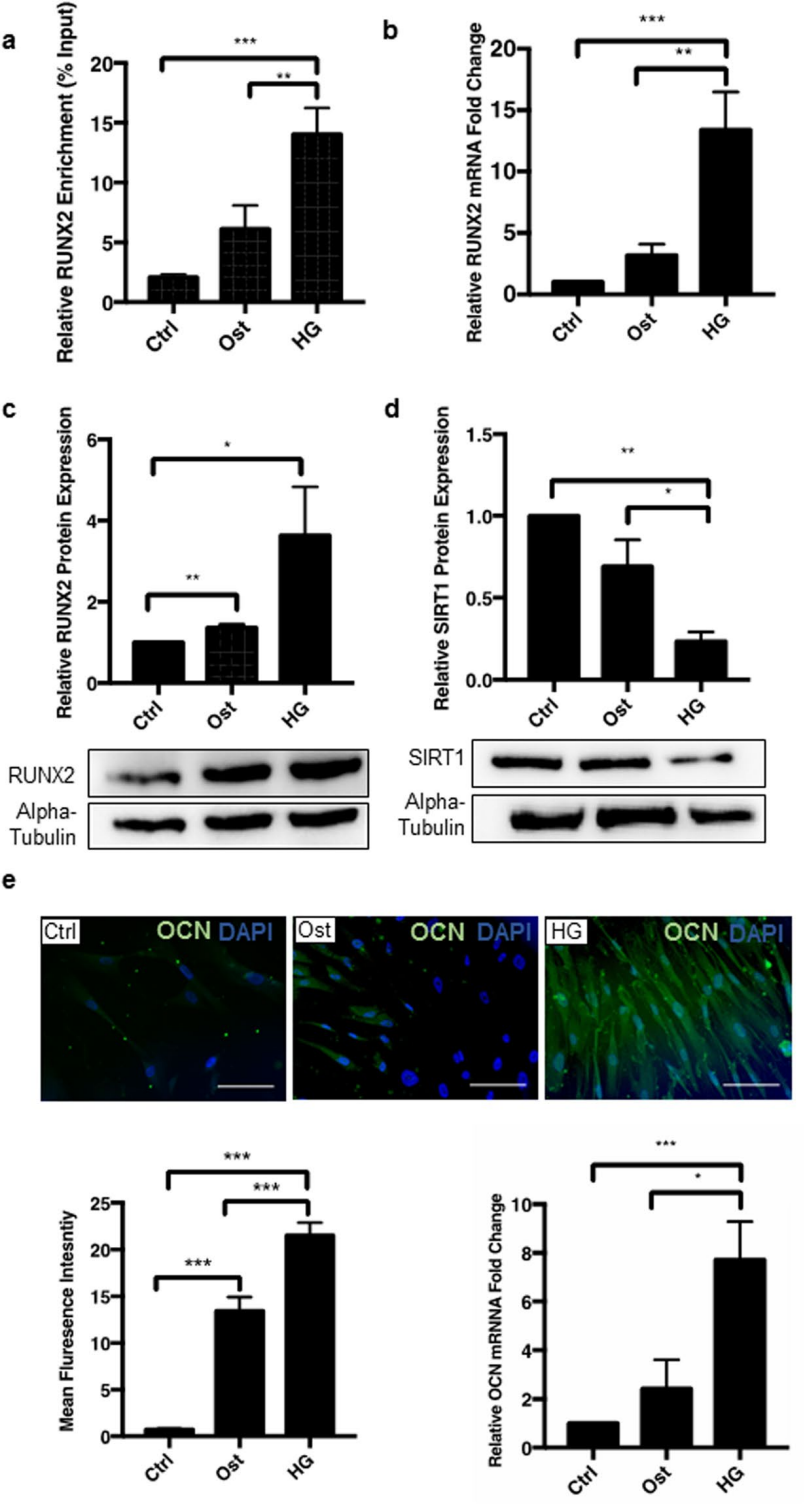
Hyperglycaemic conditions increase RUNX2 promotor acetylation and protein expression of downstream markers. (**a**) Chromatin immunoprecipitation demonstrated a significant increase in acetylation of the RUNX2 promotor in hyperglycaemic conditions compared to both osteogenic and control (n = 3). (**b**) RUNX2 mRNA abundance was increased by over 10-fold in hyperglycaemic conditions compared to osteogenic and untreated controls (n = 9). (**c**) RUNX2 protein levels doubled in hyperglycaemic conditions compared to untreated controls, α-Tubulin shown as loading control (n = 3). (**d**) SIRT1 protein levels were significantly decreased by day 4 under hyperglycaemic conditions, compared to osteogenic conditions at day 4, with α-Tubulin as a loading control (n = 3). (**e**) OCN protein level assessed by fluorescent staining shown in green, increased by a third in hyperglycaemic conditions compared to osteogenic conditions (n = 4 and 5FoV). OCN mRNA abundance increased four-fold in hyperglycaemic conditions compared to osteogenic (n = 9). *P < 0.05, **P < 0.005, ***P < 0.001. Scale Bars = 10 μm.

The mineralisation process was also confirmed at a protein level, showing a significant increase in another osteogenic transcription factor RUNX2, known to be an early marker of osteogenesis. Since RUNX2 promoter acetylation is known to regulate *Runx2* expression leading to osteogenic differentiation, the effect of SIRT1 on this process was investigated using chromatin immunoprecipitation (ChIP), using an IgG control to check efficiency (Supplementary Fig. S3). In hyperglycaemic conditions, acetylation of the promotor region was significantly increased compared to osteogenic (p < 0.0002) and control conditions (p < 0.0064) suggesting an increase in RUNX2 production during osteogenic differentiation (Fig. 3a). Furthermore, hyperglycaemic conditions increased RUNX2 mRNA significantly compared to control (p < 0.0002) and osteogenic conditions (p < 0.0057) (Fig. 3b), as well as increasing RUNX2 protein levels two-fold (p < 0.0232) in hyperglycaemic conditions compared to the untreated control (Fig. 3c). Given that SIRT1 expression was downregulated within diabetic serum compared to healthy control serum, the role of hyperglycaemic and osteogenic conditions on SIRT1 expression was examined in vSMCs *in vitro*. SIRT1 protein levels were significantly decreased in hyperglycaemic conditions by day 4, compared to cells in the control (p < 0.0001) and osteogenic conditions (p < 0.0255), demonstrating that SIRT1 is reduced during vascular smooth muscle calcification *in vitro* (Fig. 3d).

Since the gene encoding the non-collagenous bone matrix protein, osteocalcin (OCN) is a downstream target of RUNX2 and is known to have strong links with vascular calcification^34^, OCN mRNA was assessed during the osteogenic differentiation of vSMCs. OCN mRNA was significantly elevated in hyperglycaemic conditions compared to both control (p < 0.0004) and osteogenic conditions at day 4 (p < 0.0209). This result was strengthened by immunofluorescent cell staining, where OCN protein staining was also significantly increased in hyperglycaemic conditions compared to osteogenic treatment (p < 0.0001) (Fig. 3e). Having confirmed an inverse correlation between hyperglycaemia-induced mineralisation and SIRT1 levels, the direct effects SIRT1 in vSMC phenotypic changes under diabetic conditions was further investigated via pharmacological manipulation to activate and inhibit SIRT1 activity.

### Pharmacological activation of SIRT1 has protective effects against calcification

**Figure 4:**
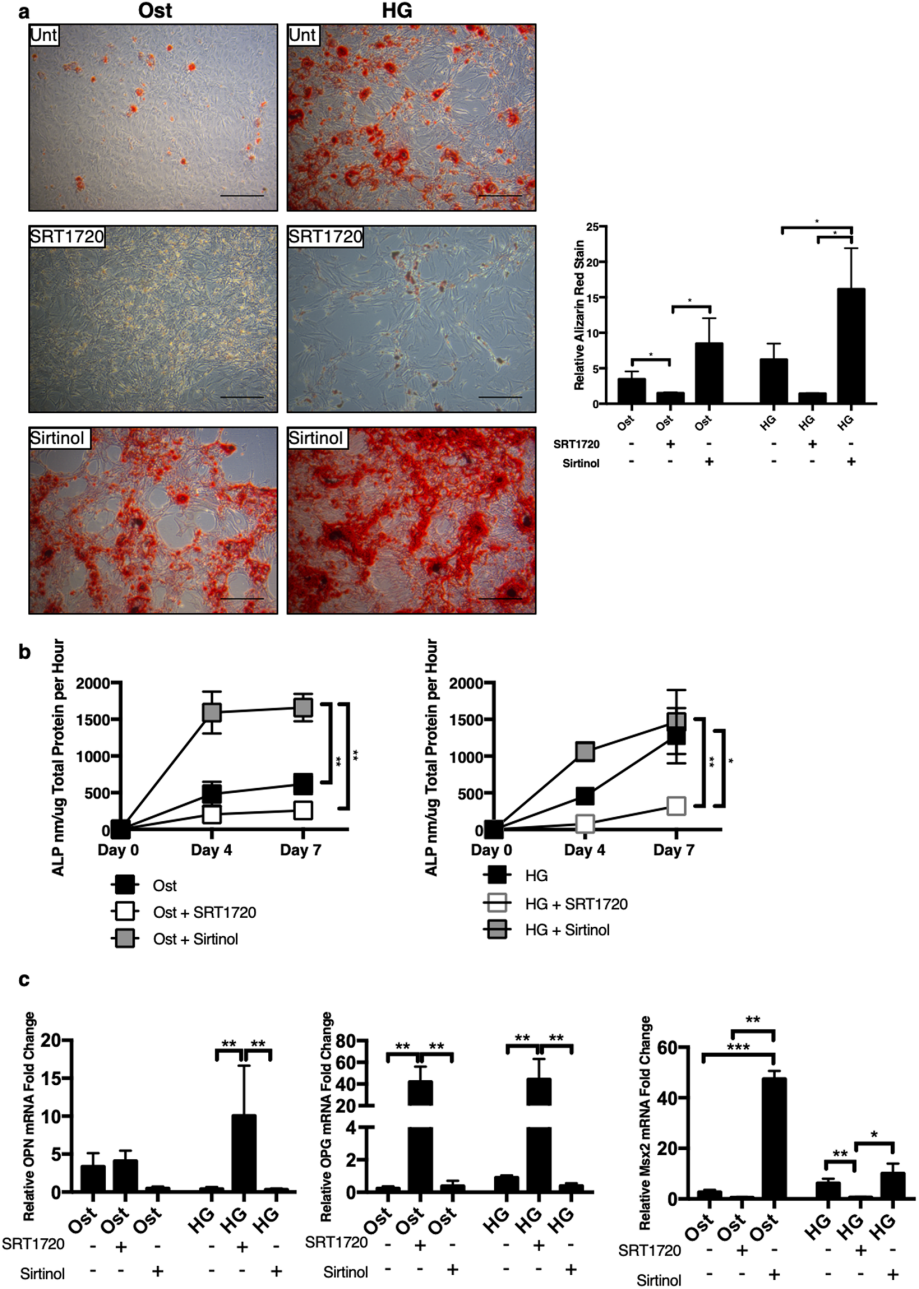
Activation of SIRT1 relives smooth muscle cell mineralisation. The effect of SIRT1 activator SRT1720 and SIRT1 inhibitor Sirtinol on smooth muscle cell calcification was investigated. (**a**) Representative micrographs show positive Alizarin red staining under untreated hyperglycaemic conditions, which was inhibited via addition of SRT1720 and exacerbated via Sirtinol, Alizarin red staining was significantly increased in Sirtinol staining by day 21 (n = 4 and 5FoV). (**b**) SIRT1 activator decreased ALP activity in both osteogenic and hyperglycaemic conditions, compared to SIRT1 inhibitor treatment (n = 3–5). (**c**) SIRT1 activation increased OPN expression in hyperglycaemic conditions, whereas Sirtinol inhibited OPN mRNA expression. OPG mRNA expression was significantly increased in both osteogenic and hyperglycaemic conditions with SIRT1 activation, and significantly decreased when SIRT1 was inhibited. MSX2 was significantly increased in both osteogenic and hyperglycaemic conditions where SIRT1 was inhibited compared to SIRT1 activation and control conditions. (n = 3). *P < 0.05, **P < 0.005. Scale Bars = 100 μm.

Cell matrix mineralisation was examined under the different treatments via Alizarin red staining. SIRT1 activation via SRT1720, protected cells from calcification in both osteogenic and hyperglycaemic conditions, as shown by the reduction of Alizarin red staining (p < 0.0492) (Fig. 4a). In contrast, SIRT1 inhibition via Sirtinol, induced a more pronounced calcium deposition in both osteogenic (p < 0.0322) and in hyperglycaemic conditions (p < 0.0332) (Fig. 4a). Control cells, treated with either SRT1720 or Sirtinol, did not show any positive Alizarin red staining (Supplementary Fig. S2). To further confirm the calcification process following SIRT1 modulation, assessment of ALP activity, was performed. Activation of SIRT1 showed a reduction in ALP activity at day 7, in both osteogenic and hyperglycaemic (p < 0.0303) conditions, compared to the untreated cells (Fig. 4b). In contrast, SIRT1 inhibition increased ALP activity at day 7, in both osteogenic (p < 0.0038) and hyperglycaemic conditions.

To add strength to the anti-calcification effects of SIRT1, additional bone-related markers were analysed (Fig. 4c). OPN mRNA abundance was significantly increased with SIRT1 activation (SRT1720) under hyperglycaemic conditions (p < 0.094), as was OPG at both osteogenic (p < 0.0046) and hyperglycaemic (p < 0.0035) conditions. Likewise, inhibition of SIRT1 significantly reduced both OPN (p < 0.0088) and OPG (p < 0.0052) compared to SRT1720 treated cells. MSX2 was significantly increased in both osteogenic (p < 0.0001) and hyperglycaemic (p < 0.0296) conditions when SIRT1 was inhibited, with the contrary true for SIRT1 activation (p < 0.0081).

**Figure 5:**
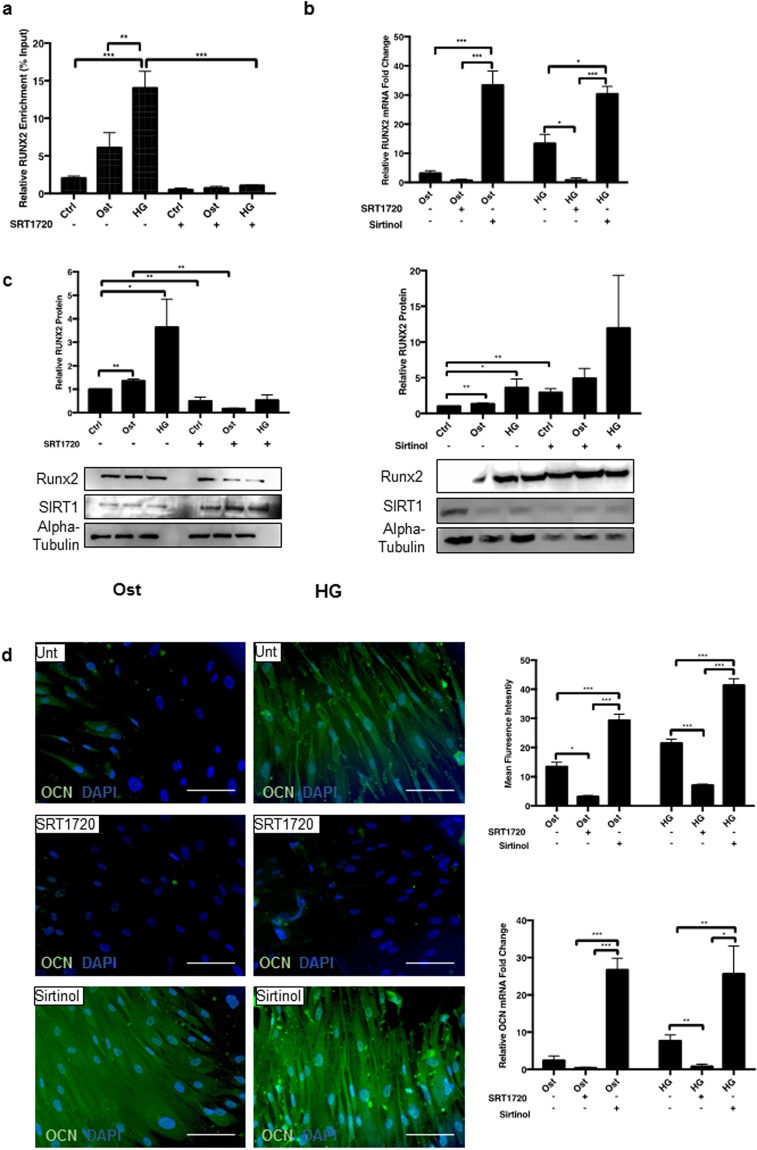
Activation of SIRT1 relives RUNX2 and Osteocalcin activation. (**a**) RUNX2 mRNA significantly decreased via SRT1720 activation and significantly increased via Sirtinol treatment (n = 4–9). (**b**) Acetylation of RUNX2 promoter region was determined via chromatin immunoprecipitation. RUNX2 promotor acetylation was significantly reduced with SRT1720 activation (n = 3). (**c**) RUNX2 protein was significantly decreased via SRT1720 activation and significantly increased via Sirtinol treatment (n = 3). (**d**) OCN activity measured via fluorescence increased in hyperglycaemic conditions compared to osteogenic, with SIRT1 activator attenuating this process and SIRT1 inhibitor Sirtinol increasing OCN fluorescence (n = 4 and 5FoV), OCN mRNA followed a similar pattern, with a decrease in OCN transcripts in SRT1720 activated cells (n = 4–9). *P < 0.05, **P < 0.005, ***P < 0.001. Scale Bars = 10 μm.

RUNX2 expression was assessed during the early phases of the differentiation process, following treatment with pharmacological manipulation of SIRT1. The acetylation profile of the RUNX2 promotor was measured via ChIP qPCR. RUNX2 promotor acetylation was significantly reduced in the hyperglycaemic conditions with the addition of SRT1720, suggesting a decrease in RUNX2 transcription was a direct result of SIRT1 activation (Fig. 5a). Furthermore, SRT1720 activation of SIRT1 activity resulted in a reduction in RUNX2 mRNA under osteogenic conditions, with a further significant reduction in RUNX2 expression under hyperglycaemic conditions (p < 0.0231), compared to the untreated cells (Fig. 5b). Conversely, Sirtinol induced a twenty-fold and a fifteen-fold increase in RUNX2 mRNA in osteogenic (p < 0.0001) and hyperglycaemic (p < 0.0355) conditions respectively, compared to their untreated conditions (Fig. 5b). The decrease in RUNX2 mRNA expression after SIRT1 activation correlates with a decrease in RUNX2 protein expression in both osteogenic and hyperglycaemic conditions at day 4. Conversely, RUNX2 protein was increased following inhibition of SIRT1 (Fig. 5c). Downstream of RUNX2, OCN was reduced at both mRNA and protein levels (Fig. 5d). SRT1720 activation of SIRT1 decreased hyperglycaemic-induced OCN mRNA eight-fold (p < 0.0137) and OCN protein by two-fold, whereas SIRT1 inhibition increased both OCN mRNA (p < 0.0089) and protein expression greater than two-fold, as shown by quantification of immunofluorescence staining (p < 0.0001) (Fig. 5d). Although control cells treated with Sirtinol appeared to increase OCN protein expression, this was not found to be significant (Supplementary Fig. S4).

### Suppression of SIRT1 results in cellular senescence under hyperglycaemic conditions *in vitro*

**Figure 6:**
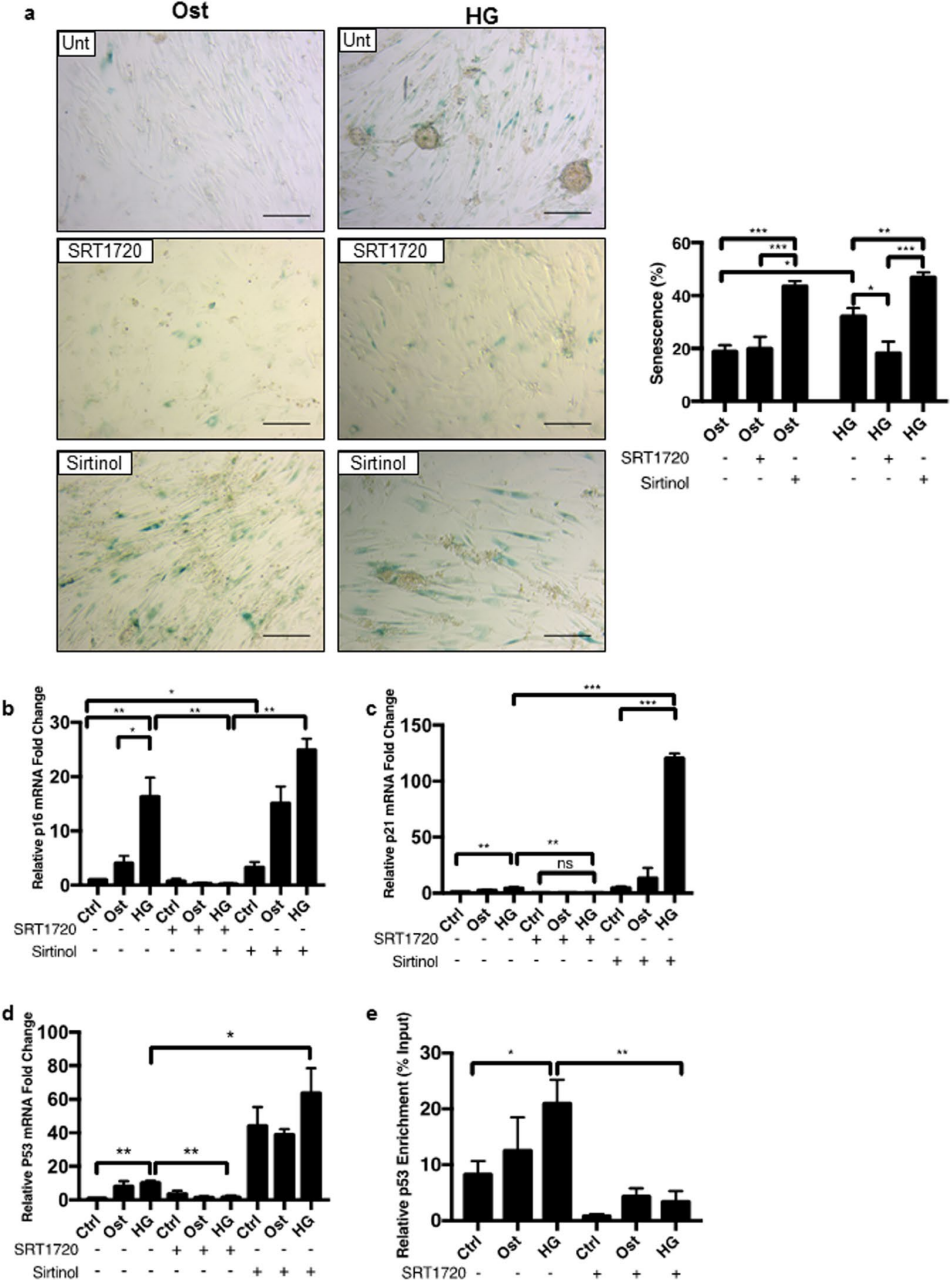
Hyperglycaemic conditions increase senescence which is modulated via SIRT1 activity. The role of SIRT1 in hyperglycaemic induced senescence was examined. (**a**) Positive cellular senescence represented via blue β-galactosidase activity seen in representative micrographs. Senescence increased significantly day 4 in osteogenic conditions, with a further significant increase in the hyperglycaemic conditions. SRT1720 activation of SIRT1 significantly decreased this trend, whereas Sirtinol induced inhibition of SIRT1 significantly increased senescence in all treatments (n = 4 and 5FoV). (**b**) p16 mRNA expression was increased in osteogenic conditions and further increased in hyperglycaemic conditions compared to control untreated cells, whereas SIRT1 activation significantly decreased p16 expression, and Sirtinol significantly increased levels of p16 transcript (n = 4). (**c**) p21 mRNA abundance was also observed. Levels of p21 transcript significantly increased in hyperglycaemic conditions, further enhanced via SIRT1 inhibition, with a significant reduction detected in SRT1720 treated cells (n = 4). (**d**) p53 mRNA expression was significantly increased during hyperglycaemic treatment and further enhanced with SIRT1 inhibition. SIRT1 activation significantly reduced p53 mRNA production. (n = 4) (**e**) Chromatin immunoprecipitation was performed with an anti-acetyl-lysine antibody. Acetylation of the p53 promotor region increased during hyperglycaemic treatment, which was reversed with the addition of SIRT1 activator SRT1720 (n = 3). *P < 0.05, **P < 0.005, ***P < 0.001. Scale Bars = 100 μm.

Since SIRT1 has been widely reported to increase lifespan in yeast, improve viability in *in vitro* cell models^35,36^, act to protect against vSMC calcification in animal models^24,37^, and its loss has been linked to premature DNA damage and senescence^37^, the effect of SIRT1 loss in a hyperglycaemic model was investigated on cellular senescence in human vSMCs using the β-galactosidase assay. Senescence was evident at day 4 with an inverse correlation (p < 0.0359) (r^2^ = 0.2469) between SIRT1 levels and the presence of senescent cells (Supplementary Fig. S5). Furthermore, there was a significant increase in the level of cellular senescence under osteogenic conditions compared to untreated control cells (p < 0.0417), and a further increase in the numbers of SA-βgal positive cells when exposed to hyperglycaemic osteogenic conditions, compared to the osteogenic conditions (p < 0.0094) (Fig. 6a). Control SRT1720 only treated cells showed no positive staining, however control cells treated with Sirtinol showed an increase in positive staining (Supplementary Fig. S6). Senescence was attenuated following SIRT1 activation by SRT1720, in both osteogenic and hyperglycaemic conditions (p < 0.0262), whereas senescence was increased following SIRT1 inhibition by Sirtinol in both osteogenic and hyperglycaemic treatments (p < 0.0022) compared to untreated controls (Fig. 6a).

Senescent cells lose their capacity to proliferate, due to an irreversible cell cycle arrest, therefore, the expression of cyclin-dependent kinase inhibitor, p16, which controls cell transition from G1 to S phase, was investigated at day 4, when senescence was apparent^38^. p16 mRNA was increased by 16.3-fold under hyperglycaemic conditions compared to osteogenic treatment (p < 0.0374) (Fig. 6b) with a significant decrease observed when SIRT1 was activated (p < 0.0040). SIRT1 inhibition caused an increase in p16 mRNA in osteogenic conditions (p < 0.0303) and hyperglycaemic conditions, when compared to controls (Fig. 6b).

p21 is a major target of p53 activity and associated with DNA damage, which is known to be involved in the pathological regulation of calcification^39^. p21 mRNA was increased 4.3-fold in cells under hyperglycaemic conditions (p < 0.0018), compared to untreated controls (Fig. 6c) and this was significantly increased in SRT1720 treated cells (p < 0.0048) and significantly increased in vSMCs, when SIRT1 was inhibited (p < 0.0001) in hyperglycaemic conditions compared to untreated cells. The increase in p21 mRNA also correlated with an increase in p16 mRNA expression (p < 0.0106) (r2 = 0.393) (Supplementary Fig. S7), which in turn, was associated with a significant increase in senescence and calcification of cells *in vitro* under hyperglycaemic conditions.

Upstream of p21, p53 mRNA was also increased in all treatments where SIRT1 is inhibited (p < 0.0232) (Fig. 6d). Conversely, p53 was reduced when SIRT1 was activated (p < 0.0079), Acetylated lysine surrounding the p53 promotor was significantly upregulated in hyperglycaemic conditions compared to control (p < 0.0188) (Figs 6e and S8) and significantly decreased with the addition of SIRT1720 (p < 0.0027), reducing the overall activation of p53.

### Suppression of SIRT1 induces early development of calcification

**Figure 7:**
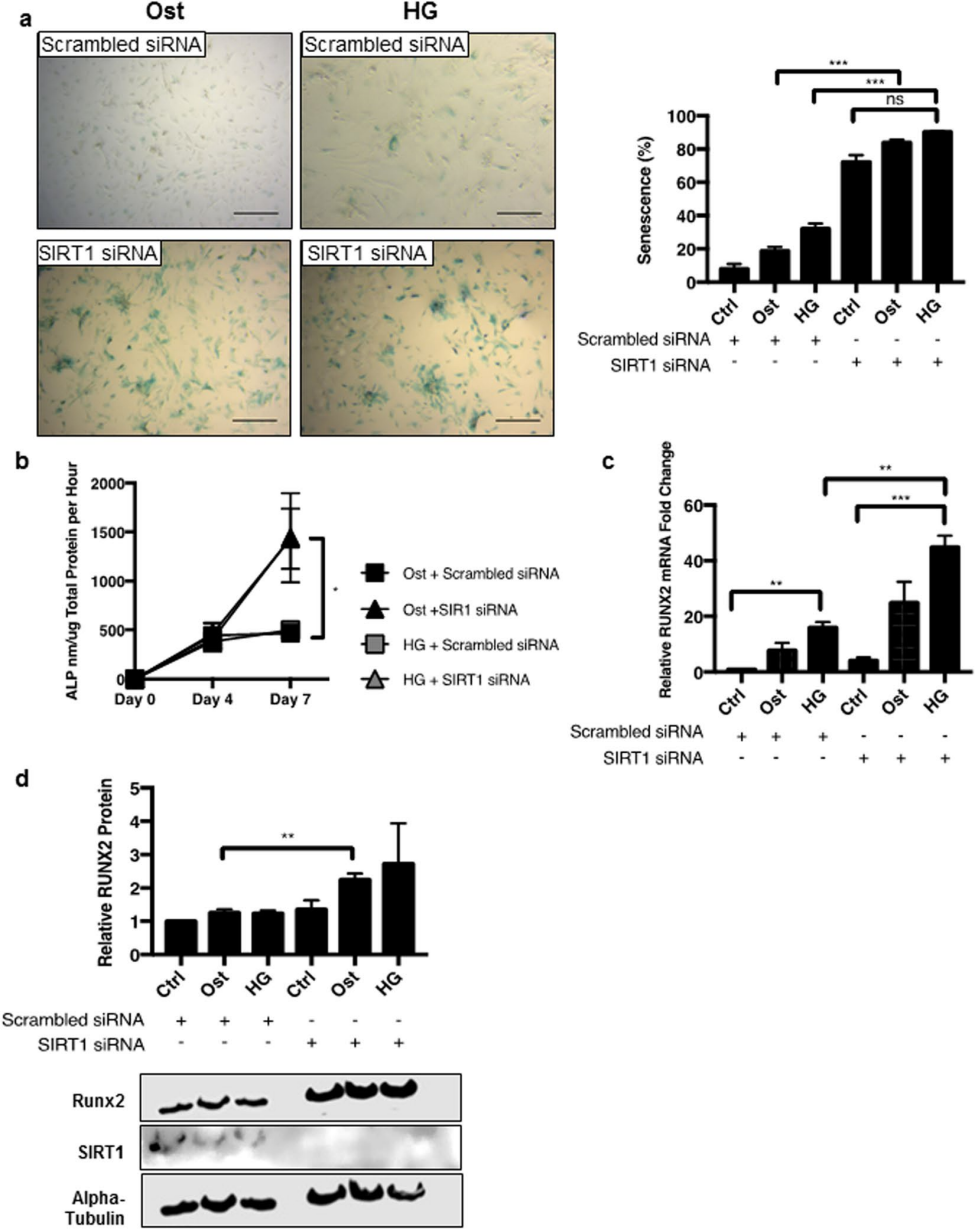
Transient ablation of SIRT1 via siRNA enhances SMC mineralisation *in vitro*. (**a**) Positive senescence associated β-galactosidase staining shown in blue in representative micrographs. Senescence increased by over 40% within four days, however no difference was detected with hyperglycaemic treatment (n = 4 and 5FoV). (**b**) ALP activity is significantly increased by day 7 in both osteogenic and hyperglycaemic treatments (n = 4). (**c**) Increased RUNX2 mRNA fold change was observed in SIRT1 siRNA treated cells compared to scrambled siRNA, with an increase observed in both osteogenic and hyperglycaemic conditions (n = 4). (**d**) RUNX2 protein levels significantly increased by day 4 in SIRT1 siRNA osteogenic conditions compared to scrambled siRNA controls (n = 3). *P < 0.05, **P < 0.005, ***P < 0.001. Scale Bars = 100 μm.

To validate the pharmacological effects of SIRT1 on the calcification process, siRNA knockdown of SIRT1 was utilised and inhibition of SIRT1 was sustained for up to seven days (Supplementary Fig. S9). Increased SA-βgal-positive staining was observed in both osteogenic (p < 0.0001) and hyperglycaemic (p < 0.0001) conditions following SIRT1 siRNA treatment, compared to respective scrambled siRNA controls, suggesting increased senescence following SIRT1 knockdown (Fig. 7a) (Supplementary Fig. S10). Furthermore, ALP activity, another early marker of osteogenic differentiation of vSMCs, was significantly increased in SIRT1 siRNA-treated cells at day 7 in hyperglycaemic conditions, when compared to the scrambled controls (p < 0.0327) (Fig. 7b). SIRT1 siRNA treatment also elevated RUNX2 mRNA abundance compared to scrambled siRNA controls in hyperglycaemic conditions (p < 0.0033) (Fig. 7c), supporting the data generated by the pharmacological inhibition with Sirtinol treatment (Fig. 4a). Likewise, RUNX2 protein levels were significantly increased in osteogenic conditions (p < 0.0092), with an increasing trend in the hyperglycaemic conditions when compared to the scrambled controls (Fig. 7d).

## Discussion

This is the first study linking the loss of SIRT1 signalling with senescence and osteogenic differentiation in human vSMCs and presence of calcification in diabetic tissue, supporting the hypothesis that a suppression of SIRT1 orchestrates the osteogenic trans-differentiation of smooth muscle cells, via induction of senescence. The senescent and osteogenic pathways and the subsequent process of mineralisation are dependent on the presence of inorganic phosphate and hyperglycaemic conditions *in vitro*, reflective of the diabetic micro-environment *in vivo*. SIRT1 polymorphisms have been linked with abnormal cholesterol metabolism, coronary artery calcification^40^ and down-regulation of SIRT1 has been shown to enhance calcification of rat SMCs *in vitro*^24^, however, the underlying mechanisms remain obscure. This study aimed to address the crucial role that SIRT1 plays in protecting against human soft tissue calcification in a diabetic context.

Firstly, this study clearly demonstrates a significant suppression of SIRT1 in serum from diabetic patients compared to healthy controls, potentially accounting for the prevalence of vascular calcification in patients with diabetes. Histological staining also shows a localisation of SIRT1 expression within the medial vSMC layer in uncalcified IMA tissue compared to the lack of positive staining in diabetic tissue samples. The development of calcification in the diabetic tissue is further supported by the evidence of RUNX2 and OCN protein localised within the diabetic vSMC layer, which is absent in the non-diabetic IMA control. Taken together, this data demonstrates a correlation between the suppression of SIRT1 and activation of RUNX2, as contributing factors to osteogenic differentiation in human tissue, suggesting the loss of SIRT1 orchestrates the osteogenic trans-differentiation of smooth muscle cells and that SIRT1 down-regulation facilitates the progression of vascular calcification in diabetes. Interestingly, RUNX2 activation in osteoblasts has been demonstrated to be positively regulated by SIRT1^41^, highlighting the differential regulatory properties in different cell types.

To further investigate the mechanistic pathways involved, human coronary artery vSMCs were grown in osteogenic conditions in the presence of high glucose, to determine how SIRT1, or its absence, mediates smooth muscle calcification. Calcification was confirmed using previously reported markers of vSMC osteogenic differentiation, including Alizarin red staining and OPN, OPG, MSX2, RUNX2 and OCN upregulation^28,42–44^. Alkaline phosphatase activity, an enzyme known to induce calcification via the depletion of the potent calcification inhibitor, pyrophosphate^19^, is recognised as an early response to the pathological stimuli used in this study, namely elevated calcium, phosphate and glucose. ALP activity was shown to be elevated in osteogenic conditions compared to control cells, with a further increase in ALP activity in the presence of hyperglycaemic conditions. Additionally, RUNX2, OCN and Alizarin red staining were all upregulated in the osteogenic conditions, and further upregulated in the hyperglycaemic conditions, suggesting that hyperglycaemic conditions, which are manifested in diabetes, are likely to contribute the development of calcification, and that SIRT1 activation may inhibit this development^23,45^. Whilst the role of OPG and OPN in calcification are controlled in a spatial and temporal manner dependant on the disease microenvironment^46^, the upregulation of both OPG and OPN in response to SIRT1 activation that was shown in this *in vitro* study, suggests that they may act as calcification inhibitors and contribute to the protective effects afforded by SIRT1^47–49^, thus suggesting that SIRT1 may play a role in the OPN-OPG axis.

SIRT1 is a member of the sirtuin family, which has previously been implicated in a range of both histone and non-histone target protein deacetylation, including age-associated senescence^50^, which in turn, has been linked to a range of pathological processes^51^, from stress response to aging to metabolic deregulation^52^. Pharmacological activation and suppression of SIRT1 using SRT1720 and Sirtinol respectively, showed that activation of SIRT1 caused an abrupt inhibition of the calcification process, with these findings being the first to confirm that SIRT1 activation inhibits hyperglycaemic-induced osteogenesis in a human smooth muscle model, supporting SIRT1 protective data from previous endothelial and animal studies^23,53^. The role of SIRT1 in the osteogenic differentiation process was further validated by the genetic knockdown and chemical inhibition of SIRT1, with ALP activity and RUNX2 expressed at a greater level in the absence of SIRT1, compared to the untreated conditions, suggesting that SIRT1 remains minimally present in the hyperglycaemic conditions and that this modulation is enough to determine the vSMCs osteogenic fate. This study supports and extends previous findings performed in rat cells and bone cells^23,54^, demonstrating that the loss of SIRT1 activity is crucial for the modulation of osteogenic factors. Although these data demonstrate the unique cellular adaptive responses that SIRT1 provides, the exact mechanism still remains unclear.

Since this study and others have demonstrated an inverse correlation between the diabetic environment and reduced SIRT1 levels, a high glucose *in vitro* model was used to compare the direct effect of hyperglycaemia vs osteogenic conditions on SIRT1 production. SIRT1 expression was shown to be reduced under osteogenic conditions, but notably levels were further diminished in hyperglycaemia, as calcification became more apparent. Increased glucose concentration within the blood has been shown to produce pseudohypoxia within the patient, with glucose being quickly metabolised along the sorbitol pathway^55^. Firstly, glucose is reduced to sorbitol by aldose reductase, whilst NADPH is simultaneously oxidised to NADP^+^. Following this, sorbitol is then oxidised by sorbitol dehydrogenase to fructose, reducing NAD^+^ to NADH, producing an increased concentration of NADH in the blood stream. This direct lack of NAD^+^ by hyperglycaemia, is a rate limiting step for SIRT1 activity, as without this co-factor, SIRT1 is unable to deacetylate its targets. As RUNX2 contains 10 conserved lysine residues which are sites of acetylation, it is likely that osteogenic growth factors such as BMPs^56^ increase acetylation, stability and transcriptional activity of Runx2 via increased p300 protein levels and histone acetyltransferase activity^57^. Once the SIRT1 co-factor NAD^+^ is diminished via hyperglycaemia it may be unable to competitively inhibit the acetylation of RUNX2, allowing an increase of calcification within the patient.

Acetylation of the RUNX2 promotor was increased in a hyperglycaemic environment, compared to both the osteogenic and control conditions. The increase in RUNX2 promoter acetylation correlated with a down-regulation of SIRT1 expression within the cells, suggesting that RUNX2 activation has a direct effect of reducing SIRT1 in vSMCs and contributing to their osteogenic differentiation. This hypothesis was proven to be correct when SRT1720 activation of SIRT1 diminished RUNX2 promoter acetylation to control levels, which in turn, correlated with a lack of RUNX2 protein at the same time point. These data demonstrate for the first time a change in the acetylation profile of RUNX2 in a diabetic model, suggesting a pathway in which SIRT1 directly influences the activation of osteogenic differentiation.

Osteogenic differentiation of vSMCs follows a distinct change in phenotype, firstly switching from a contractile phenotype to one with an increased senescent capacity, before manifesting as an increase in arterial stiffness and a decrease in arterial compliance^58^. Therefore, whilst this study demonstrates the correlation between calcification and a reduction in SIRT1, it was important to establish whether this was dependant on a senescence pathway. SIRT1 has a wide array of targets which modulate the ‘senescence-associated secretory phenotype’ protein^59^, which when acetylated can translocate to the nucleus, resulting in the development of premature cellular senescence. Pharmacological and genetic depletion of cellular SIRT1 was shown to significantly increase senescence, regardless of hyperglycaemic and phosphate treatment, suggesting a key role of SIRT1 in senescence, independent of calcification. However, senescence has also been associated with phenotypic changes in smooth muscle cells and the development of atherosclerosis^37^. Saβ-gal staining, demonstrating cellular senescence occurred at an earlier timepoint than alizarin red staining, suggesting senescence develops prior to calcification. It may be that this down-regulation of SIRT1 triggers the development of senescence, which in turn activates the smooth muscle differentiation to an osteogenic phenotype. This is supported by the lower expression of RUNX2 at day 4 under normal glucose conditions compared to hyperglycaemic conditions, suggesting that RUNX2 activation occurs more readily in a senescent environment. These findings strengthen previous studies that suggest vSMC senescence is a transient phenotype, increasing sensitivity of these cells to begin the induction of calcification^37^^,^^38^.

Since both the induction of senescence and expression of SIRT1 are commonly associated with the p21 and p16 cell cycle and proliferative pathways, the effect that SIRT1 has on their expression was investigated. Both p53 and p21 expression was found to be significantly upregulated in hyperglycaemic conditions, where SIRT1 was diminished, and then further upregulated when SIRT1 was inhibited completely. It has previously been shown that p53 acetylation is required to induce the transactivation of p21^60^ alongside other senescence markers, and that the deacetylation of p53 by SIRT1 reduces its half-life and stabilisation^61^ thus decreasing its cellular activity. Previous studies have demonstrated that a hyperglycaemic environment increases acetylation of the p53 promotor within HUVECs^62,63^ and neurons^64^, however the effect in vSMCs is unknown. This study now demonstrates that hyperglycaemic conditions significantly increase acetylation of the p53 promotor in vSMCs, which correlates with elevated p53 and p21 mRNA abundance *in vitro*. Of note, SIRT1 activation significantly decreases lysine acetylation on the p53 promoter, reducing p53 expression and in turn contributing to the reduced senescent phenotype within the cells.

As SIRT1 is decreased in the diabetic patient, p53 is sustained in a constant state of acetylation and production, thus enhancing production of the p53 target protein, p21 within the cell. Since the SIRT1 promotor region contains two response elements to p53, it has been suggested that p53 reduces SIRT1 expression in a negative feedback loop, thus propagating p21 production and increasing senescence. As previous genome-wide association studies have consistently found that p16 is associated with diabetic risk^65^, this study examined p16 expression, which was found to be increased both under hyperglycaemic conditions and after SIRT1 inhibition. Previous studies have reported an increase in p16 production when p300 acts to acetylate the appropriate sites on the promotor^66^, findings which are supported by this study, which demonstrates a reduction of the deacetylase SIRT1 causes an increase in p16 production. These data suggest that p16 is controlled by its acetylation profile. Additionally, this study and others report hyperglycaemic conditions rapidly induce p16 expression^67^; however the mechanism by which this occurs remains to be elucidated. Based on the current findings, hyperglycaemic inhibition of SIRT1 leads to increased production of p21 and p16, thus promoting the senescent phenotype in vSMCs, and acting as a basis for calcification development.

**Figure 8:**
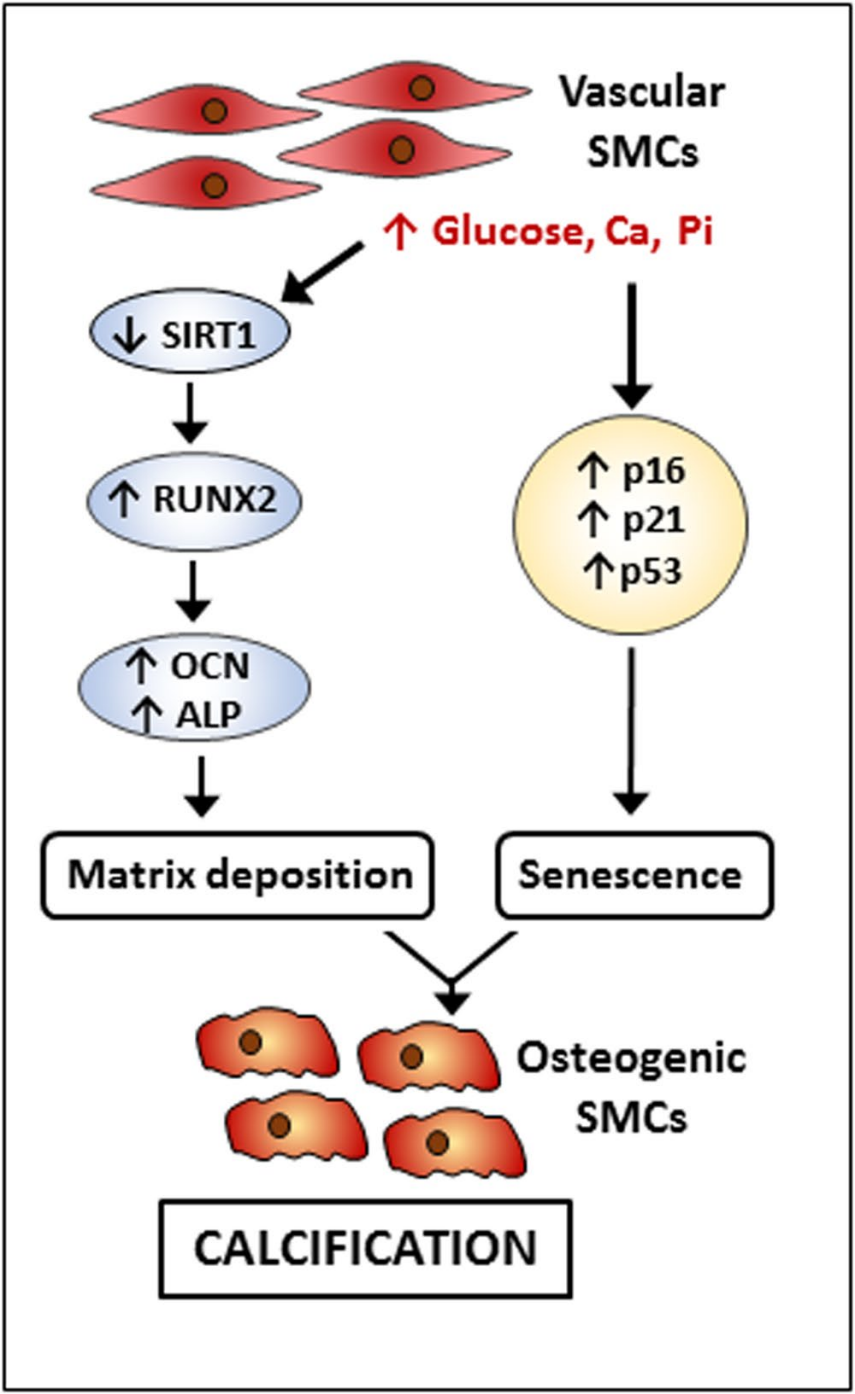
Suggested mechanism of SIRT1 within vascular calcification. High glucose, phosphate and calcium lead to a reduction in SIRT1 expression and a simultaneous increase in osteogenic markers, RUNX2, OCN and ALP alongside the induction of a senescent phenotype, exacerbating the calcification of vascular SMCs.

In conclusion, this study has demonstrated the protective effect of SIRT1 in a hyperglycaemic model of human vascular calcification *in vitro*, summarised schematically in Fig. 8, suggesting the connection between cellular senescence and calcification. We suggest that the loss of SIRT1 results in the induction of calcification which acts in synergy with the induction of senescence in the presence of high glucose, forming a transient state in which smooth muscle cells can develop calcification. Further investigations into the role of SIRT1 in cell fate determining pathways in vascular calcification are now warranted. Identification of other molecular pathways involved in the development of calcification should be investigated, to determine the global effect of the decrease of SIRT1 in both the diabetic patient and those susceptible to vascular calcification. The use of novel compounds to maintain a higher level of SIRT1 activity in newly diagnosed diabetic patients may provide a therapeutic opportunity to prevent the onset and development of calcification.

## Methods

### Cell Culture and Induction of Calcification

Primary human coronary artery vSMCs were cultured according to the manufacturer’s instructions (Caltag, UK) in SMC Basal Media 2 (PromoCell), until 80% confluent, then transferred to either; DMEM containing 5 mM glucose (control conditions), DMEM 5 mM glucose, supplemented with 5 mM β-glycerophosphate and 2.6 mM CaCl_2_ (Osteogenic conditions) or DMEM containing 25 mM glucose supplemented with 5 mM β-glycerophosphate and 2.6 mM CaCl_2_ (hyperglycaemic conditions). SRT1720^68^ and Sirtinol^69^ were used at dose dependent concentrations confirmed by cell viability assays (Supplementary Fig. S10). Cells were not used beyond passage 6. All experiments were performed in duplicate, with a minimum of three populations of cells for each experiment.

### SiRNA knockdown

Transient transfection of siRNA via lipofectamine 2000 (Invitrogen) was achieved in vSMCs. vSMCs were seeded at a density of 4 × 10^5^/cm^2^ and transfected with SIRT1 siRNA (20 nM) or scrambled control RNA (20 nM) in antibiotic free DMEM overnight before treatments. Down-regulation of SIRT1 was confirmed by Western blot analysis (Supplementary Fig. S9).

### Alizarin Red Staining and Quantification

Alizarin Red S staining was used to detect matrix mineralisation by the vSMCs as previously described^28^. Briefly, cells were cultured for 21 days, washed with PBS and fixed with 4% paraformaldehyde in PBS for 10 min at room temperature, followed by staining with 2% Alizarin Red S in H_2_O for 5 minutes. Culture plates were washed 4 times with distilled H_2_O for the duration of an hour and imaged under a light microscope, with positive staining shown in red. To quantify calcification, the alizarin red was eluted with 10% formic acid and absorbance was measured at 405 nm using a spectrophotometric plate reader (HT Synergy BioTek).

### Alkaline Phosphatase (ALP) Activity

ALP activity was assessed using the p-nitrophenol phosphate (pNPP) assay as previously described^42^. Briefly, cells were lysed with 0.05% Triton-X and quantified using a BCA protein quantification kit (Thermo, UK). Enzyme activity was determined using pNPP (10 mM) and read at 410 nm spectrophotometrically and normalised to total protein. ALP activity was expressed as absorbance in nm/mg total protein per min.

### Reverse Transcription-Quantitative Polymerase Chain Reaction

Total RNA was extracted from vSMC using TriZol (Invitrogen, USA) and purity, quality and concentration assessed by optical density at 260/280 nm (Nanodrop2000, Thermo, USA). 500 ng of each sample was reverse transcribed using the Tetro cDNA Synthesis Kit (BioLine, UK) using random hexamers, as per the manufacturer’s instructions, and diluted to a working concentration (50 ng). qPCR was performed in a reaction mixture (10 µL) containing; cDNA (3 µL), of forward and reverse primers (1 µL, 100 µM; Supplementary Table S2) and 4x SYBR Green Lo-ROX (5 µL; Bioline, UK). The qPCR was cycled on a PCR machine (StepOnePlus, Thermo, UK). The initial denaturation was carried out at 95 °C for 5 minutes, followed by 40 cycles of 95 °C for 30 seconds, 58 °C for 30 seconds and 72 °C for 30 seconds, with each sample being performed in triplicate. Relative target gene expression was calculated as a fold change compared to GAPDH and β-Actin housekeeper genes, using the 2^−ΔΔCq^ method.

### Western Blot Analysis

Western blot analysis of SIRT1 and RUNX2 was performed as previously described^42^. Briefly, whole cell lysates were quantified using a BCA protein quantification kit (Thermo, UK) according to the manufacturer’s instructions. Subsequently, total protein extract (10 µg) was separated on NuPAGE Bis-Tris Gels (Invitrogen, UK) and transferred to a PVDF membrane. The membrane was blocked in 5% skimmed milk in PBST, incubated with either anti-SIRT1, RUNX2 or alpha-tubulin antibodies (Supplementary Table S1) in blocking buffer overnight at 4 °C. The membrane was washed in PBST and incubated with either horseradish peroxidase (HRP)-labelled goat anti-rabbit, rabbit anti-mouse, or rabbit anti-goat IgGs respectively, for 2 h at room temperature, washed and developed using the Immobilon ECL ultra western HRP substrate (Millipore, USA) and imaged (ChemiDoc, BioRad). Densitometry was performed using ImageLab software (BioRad) with all samples normalised to α-tubulin.

### Chromatin Immunoprecipitation

Chromatin immunoprecipitation (ChIP) assays were performed using a SimpleChIP enzymatic chromatin IP kit (#9003; Cell Signalling Technologies, USA). Briefly, 12 × 10^6^ vSMCs were treated for four days and then cross-linked with 37% formaldehyde at a final concentration of 1% at room temperature for 10 minutes. Chromatin was treated with nuclease and sonicated. Chromatin immunoprecipitation was performed with anti-acetyl-lysine, anti-histone H3 and rabbit IgG (Supplementary Table S1). Following this, samples were reverse crossing linked, purified and quantified using RT-PCR as previously described^70^, with primers designed for RUNX2 and p53 promotor regions (Supplementary Table S2). Fold enrichment was determined via the anti-acetyl-lysine immunoprecipitation compared to the 2% input sample, using the comparative *C*_*t*_ method. Amplification compared to negative control was calculation to confirm specific binding (Supplementary Figs S3 and S8).

### Cellular Senescence

Cells were cultured in duplicate for four days in control, osteogenic or hyperglycaemic conditions and subsequently fixed with 4% paraformaldehyde in PBS. Cells were then washed with phosphate buffer (0.1 M, pH7.3) supplemented with MgCl_2_ (2 mM), before staining for senescence-associated β-galactosidase (SAβ-gal) for up to four hours at 37 °C. Culture plates were washed and imaged under a light microscope and the number of SAβ-gal-positive cells were quantified using ImageJ software.

### Enzyme Linked Immunosorbent Assay

SIRT1 was quantified in serum harvested from patients with diabetes undergoing lower limb amputation and healthy controls using an AbCam SimpleStep ELISA kit (ab171573). Serum and human tissue was obtained under informed consent (Ethics Reference 14/NW/1062) and procedures were in accordance with institutional guidelines and the Declaration of Helsinki. Serum was diluted 1:2 and added to each well and processed as per manufacturer’s instructions. The plate was read at 450 nm.

### Immunohistochemical Staining

Sections (6 µm) of the popliteal artery, taken from diabetic patients, were used for histochemical analysis. A panel of internal mammary artery (IMA) segments were used as a non-atherosclerotic control artery, lacking large calcific deposits. Arterial sections were stained with antibodies at relevant dilutions (Supplementary Table S1) and a non-immune serum (IgG) as a negative control as previously described^28^. Slides were then counterstained with nuclear fast red and imaged using a brightfield microscope (Zeiss AxioVert).

### Immunofluorescence

Cells, seeded in 8 well chamber slides, were fixed with paraformaldehyde (4%) for 15 minutes at room temperature. Following fixation, cells were blocked and permeabilized with Tween-20 (1%) in 5% goat serum in PBS and stained with antibodies at relevant dilutions (Supplementary Table S1). Slides were mounted with DAPI antifade gold (Vector, UK) and imaged using a fluorescent microscope (Zeiss AxioImager Z1). Images were quantified using ImageJ software. Fluorescent staining was completed in duplicate, with five fields of view acquired from each chamber.

### Statistical Analysis

All experiments were performed a minimum of three times using independent populations of cells and ran. All data are presented as the mean ± standard error of the mean (SEM). Sharpio-Wilk normality test was used to assess normality. 1-way ANOVA was performed with relevant post-hoc T-tests using Prism 5 software (GraphPad Software, USA). Nonsignificant differences were noted ns. p values were noted as follows: *for p < 0.05; **for p < 0.005; and ***for p < 0.001.

## Supplementary information

Supplementary Dataset 1–16

## Data Availability

All data generated and analysed during this study are included in this published article and is supplementary information files.
