# Supplementary material for: Suppression of SIRT1 in Diabetic Conditions Induces Osteogenic Differentiation of Human Vascular Smooth Muscle Cells via RUNX2 Signalling
Source: Sci Rep. 2019 Jan 29;9:878. doi: 10.1038/s41598-018-37027-2 (PMC6351547; doi:10.1038/s41598-018-37027-2)
Supplement: Supplementary file 1 — Supplementary Dataset 1–16 [file 41598_2018_37027_MOESM1_ESM.pdf]

Suppression of SIRT1 in Diabetic Conditions Induces Osteogenic Differentiation of Human Vascular Smooth Muscle Cells via RUNX2 Signalling

F Bartoli-Leonard[1], FL Wilkinson[1], A Schiro[2], F Serracino Inglott[2], M Y Alexander[1], R Weston[1]*

[1]Translational Cardiovascular Science, Centre for Bioscience, Manchester Metropolitan University, [2]Vascular Unit, Manchester NHS Foundation Trust, Manchester Academic Health Science Centre, Manchester, UK

Supplementary Material

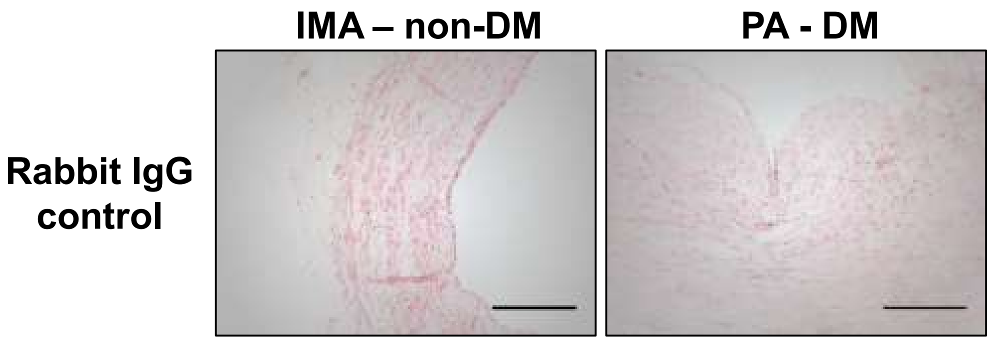

Supplementary Fig. S1
IHC Controls. Both IMA and DM popliteal artery were treated with mouse IgG as previously described[20]. Both IMA and DM sections showed no positive staining when treated with the biotintinylated secondaries used in the positive stains. Scale bar = 200 μm.

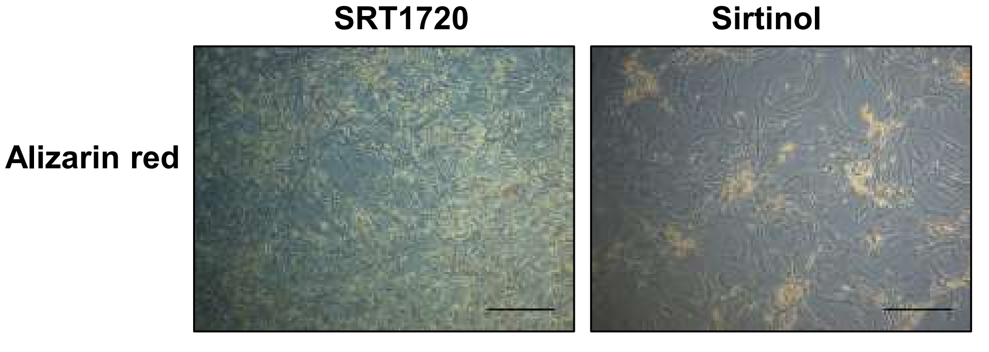

Supplementary Fig. S2
Alizarin Red staining. Representative micrographs of control (5mM glucose) treated cells with SIRT1 activator SRT1720, or SIRT1 inhibitor Sirtinol added. Positive staining observed in red was not apparent. Scale bar = 100 μm.

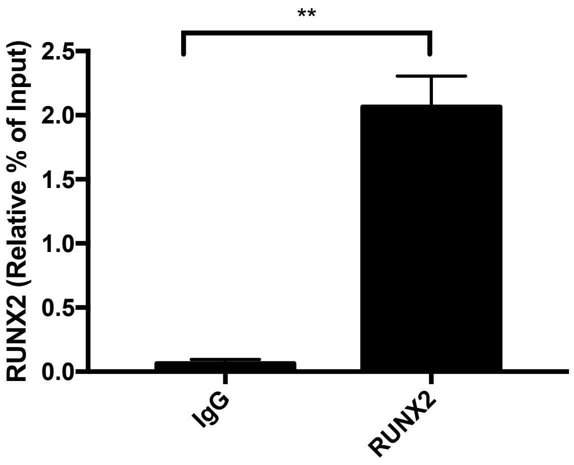

Supplementary Fig. S3
RUNX2 promotor acetylation enrichment. Acetylation of the RUNX2 promotor region was significantly upregulated when compared to the negative control; Rabbit IgG, demonstrating the fold enrichment was significant to the immunoprecipitation, and not background.

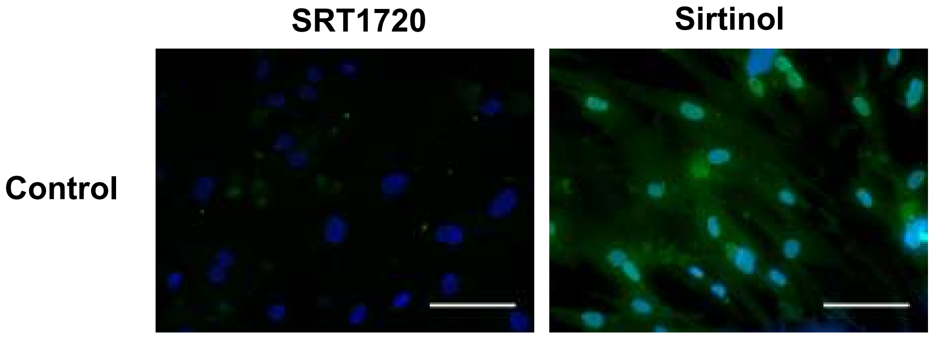

Supplementary Fig. S4
Osteocalcin immunofluorescence staining. Cells grown in control (5mM glucose) conditions were treated with SRT1720 or Sirtinol for 4 days before fixing and staining. OCN stain seen in green was not apparent in SRT1720, and very little in Sirtinol treated cells. Scale bar = 10 μm.

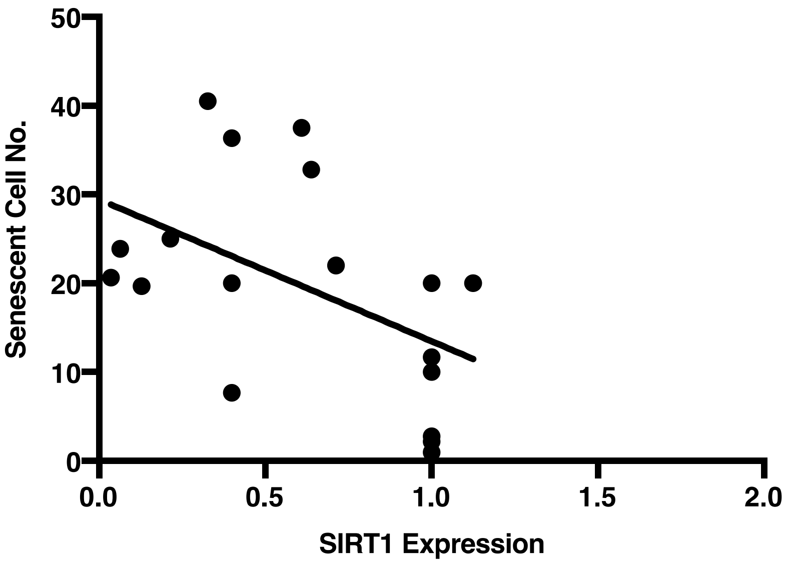

Supplementary Fig. S5
SIRT1 and B-Galactosidase Stain Correlation. Linear regression analysis was performed, comparing SIRT1 protein expression assessed from western blot data, to the number of senescent cells from the X-Gal staining. R2= 0.2469, P=0.0359.

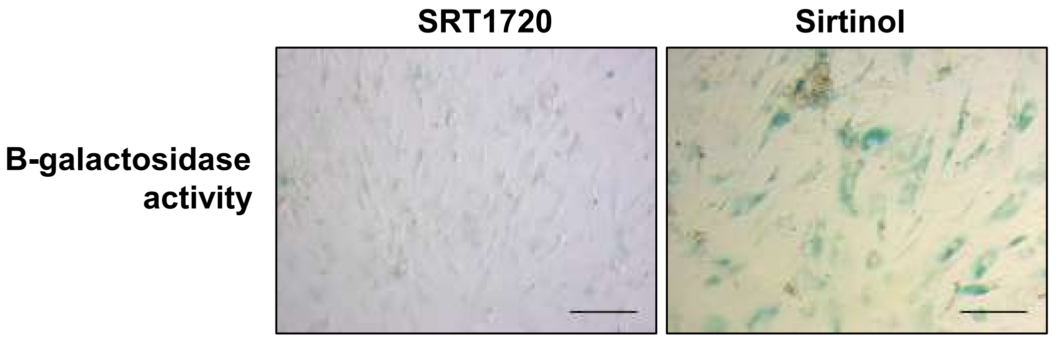

Supplementary Fig. S6
SIRT1 Modulated B-Galactosidase staining. Representative micrographs of control (5mM glucose) conditions were treated with SIRT1 activator SRT1720 and SIRT1 inhibitor Sirtinol for 4 days before staining. Control SRT1720 treated cells showed little sign of positive blue staining, whereas SIRT1 inhibited cells showed significantly greater staining. Scale bar = 100 μm.

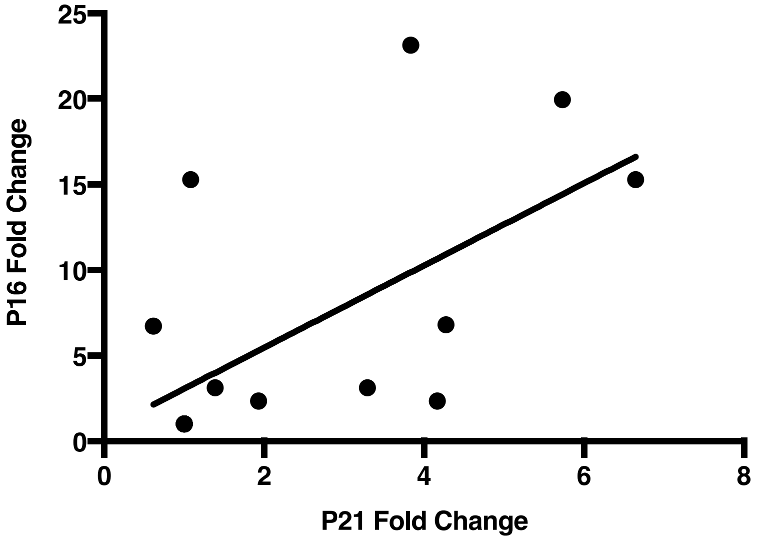

Supplementary Fig. S7

Cell Cycle Marker Correlation. Linear regression analysis was performed comparing P16 and P21 mRNA fold change values. A positive correlation is shown between the two, R2=0.383, p=0.0106

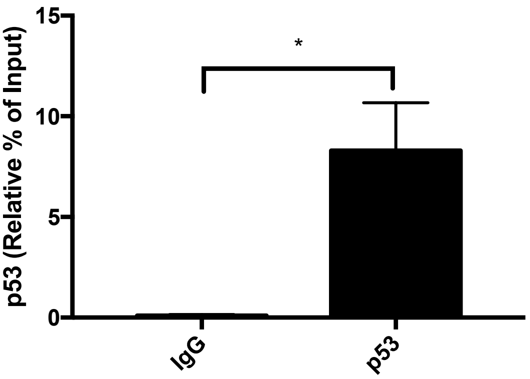

Supplementary Fig. S8

p53 promotor acetylation enrichment. Acetylation of the p53 promotor region was significantly upregulated when compared to the negative control; Rabbit IgG, demonstrating the fold enrichment was significant to the immunoprecipitation, and not background.

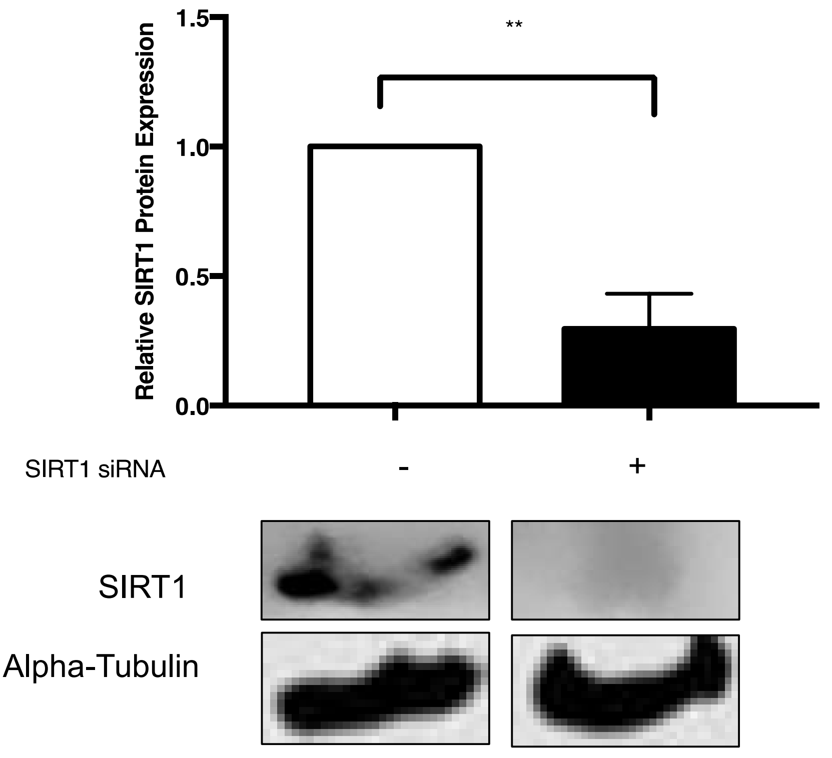

Supplementary Fig. S9.
SIRT1 sIRNA Knockdown. SIRT1 siRNA efficiency was determined via western blot. SIRT1 knockdown was also confirmed via western blot at day 7. SIRT1 siRNA decreased SIRT1 protein expression by 60% seven days after treatment.

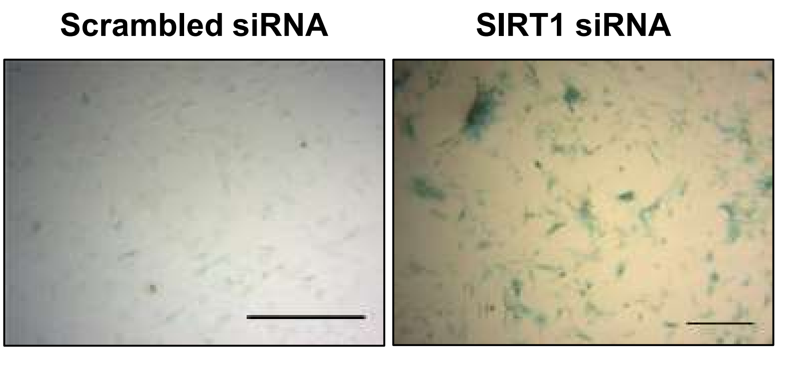

Supplementary Fig. S10.
siRNA Treated B-Galactosidase Staining. Representative micrographs of day 4 control (5mM glucose) treated scrambled siRNA or SIRT1 siRNA treated cells, stained with B-galactosidase. Significant increase in SIRT1 treated cells is observed compared to scrambled siRNA treated cells. Scale bar = 100 μm.

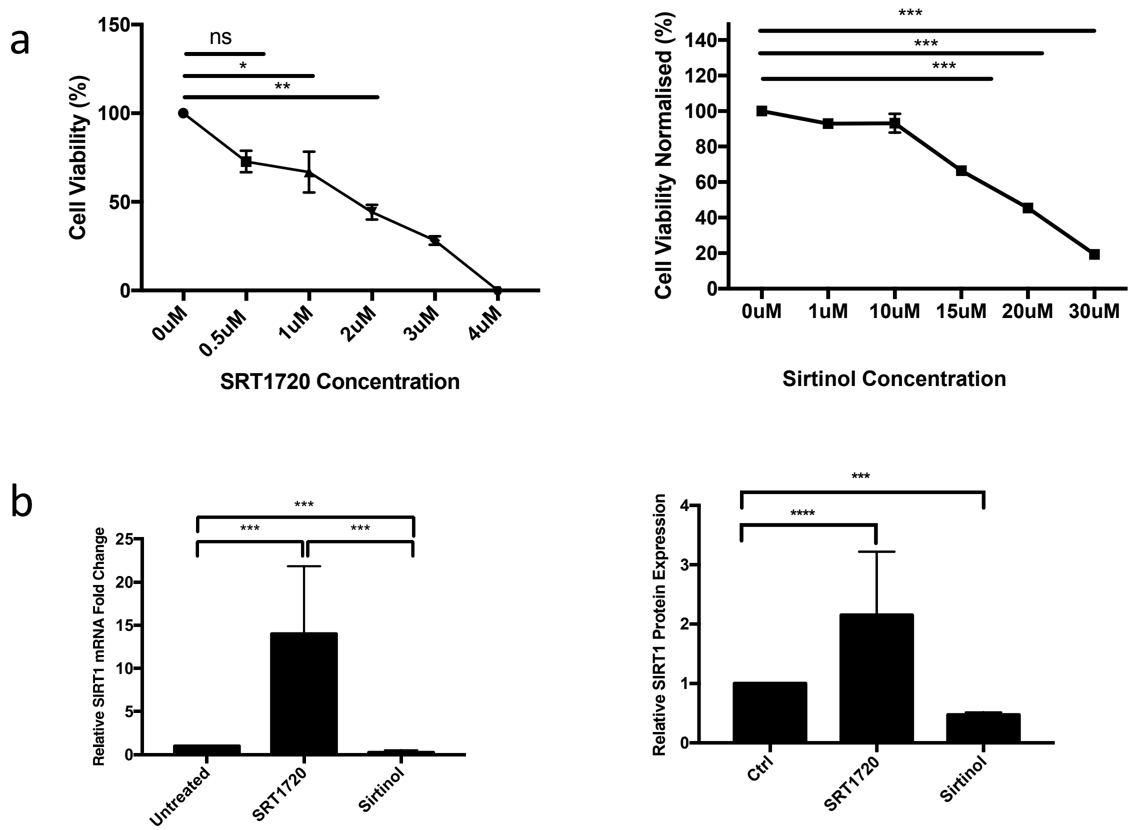

Supplementary Fig. S11.

SRT1720 and Sirtinol dose response. a) SIRT1 activator SRT1720 and inhibitor Sirtinol toxicity was determined via the AlamarBlue assay. SRT1720 did not significantly reduce cell viability at 0.5μM, and Sirtinol inhibition was not significantly toxic at 10μM. B) Cells treated for four days were then harvested for RNA and protein to determine if these doses effected SIRT1 expression. SRT1720 activation significantly increased SIRT1 expression in both mRNA and protein, whereas Sirtinol reduced SIRT1 expression to significantly less than control.

Supplementary Table S1. List of antibodies used for western blot analysis.

Antibody	Product Code	Dilution	Source
Anti-Rabbit Anti-SIRT1	ab42441	1:5000	AbCam, Cambridge, MA, USA
Goat Anti-Rabbit IgG HRP	A6154	1:2000	Dako / Agilent, Santa Clara, CA, USA
Anti-Goat Anti-RUNX2	AF2006	1:2000	R&D Systems, Minneapolis, MN, USA
Rabbit Anti-Goat IgG HRP	A9262	1:2000	Dako / Agilent, Santa Clara, CA, USA
Anti-Mouse Anti-Alpha-Tubulin	ab7291	1:5000	AbCam, Cambridge, MA, USA
Rabbit Anti-Mouse IgG HRP	A9044	1:2000	Dako / Agilent, Santa Clara, CA, USA
Anti-Rabbit Anti-Histone H3	CST4620	1:50	Cell Signalling Technology. New England, USA
Anti-Rabbit IgG	CST2729	1:500	Cell Signalling Technology, New England, USA
Anti-Rabbit Anti-Acetyl-Lysine	ab21623	1:500	AbCam, Cambridge, MA, USA

Supplementary Table S2. Sequences of primers used in real-time PCR.

Primer Name	Accession & Definition	Primer Pair Sequence 5'- 3' (Sense/Antisense)	Location (nt)	Fragment Size (bp)
SIRT1	NM_001142498.1	CTGGACAATTCCAGCCATCT<br>GGGTGGCAACTCTGACAAAT	519-1114	595
P16	NM_214646.1	CGTGGACCTGGCTGAGGAGC<br>ACGAAAGCGGGGTGGGTTGT	667-881	234
RUNX2	NM_001024630.3	GAGGGCACAAGTTCTATCTG<br>CGCTCCGGCCCACAAATCTC	192-784	592
OCN	NM_199173.5	CATGAGAGCCCTCACA<br>AGAGCGACACCCTAGAC	269-561	292
GAPDH	NM_001256799.2	CCACCCATGGCAAATTCCATG<br>TCTAGACGGCAGGTCAGGTCCACC	394-947	553
β-Actin	NM_001101.4	AGAGCTACGAGCTGCCTGAC<br>AGCACTGTGTTGGCGTACAG	2339-2598	259

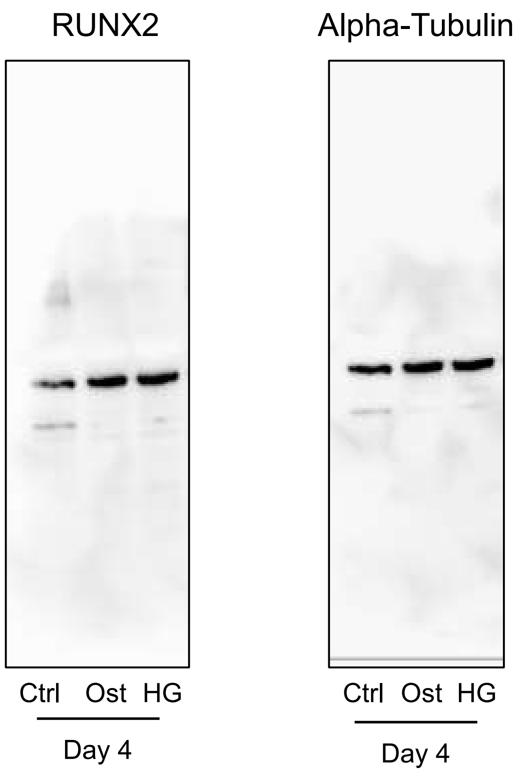

Supplementary Fig. S12.
Untreated RUNX2 Western blot

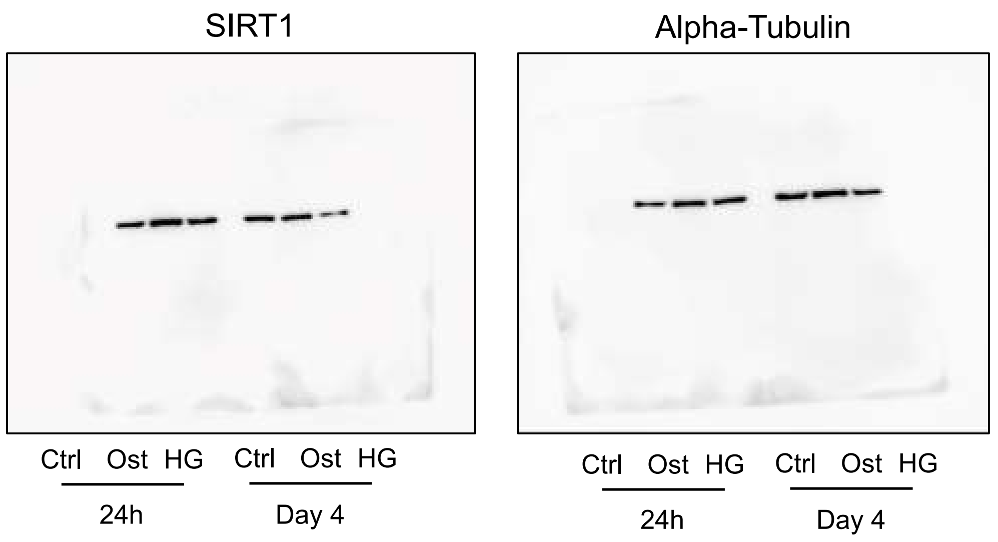

Supplementary Fig. S13.
Untreated SIRT1 Western blot

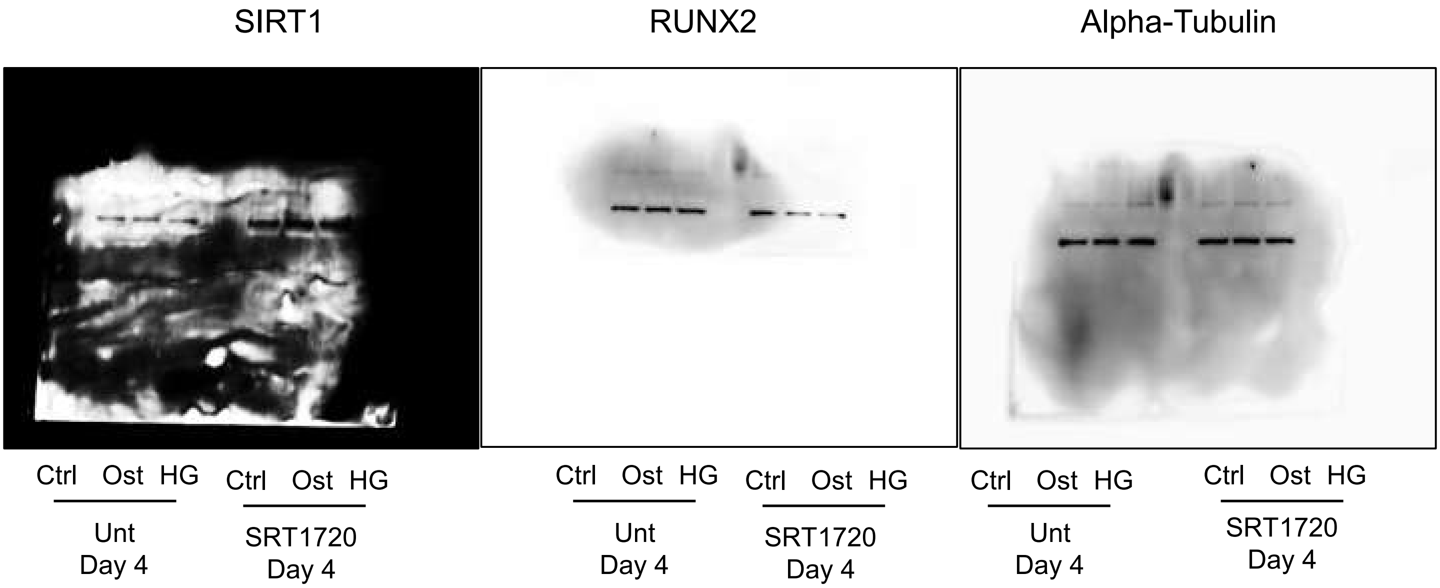

Supplementary Fig. S14.
Untreated & SRT1720 SIRT1, RUNX2 and Alpha-Tubulin Western blot.

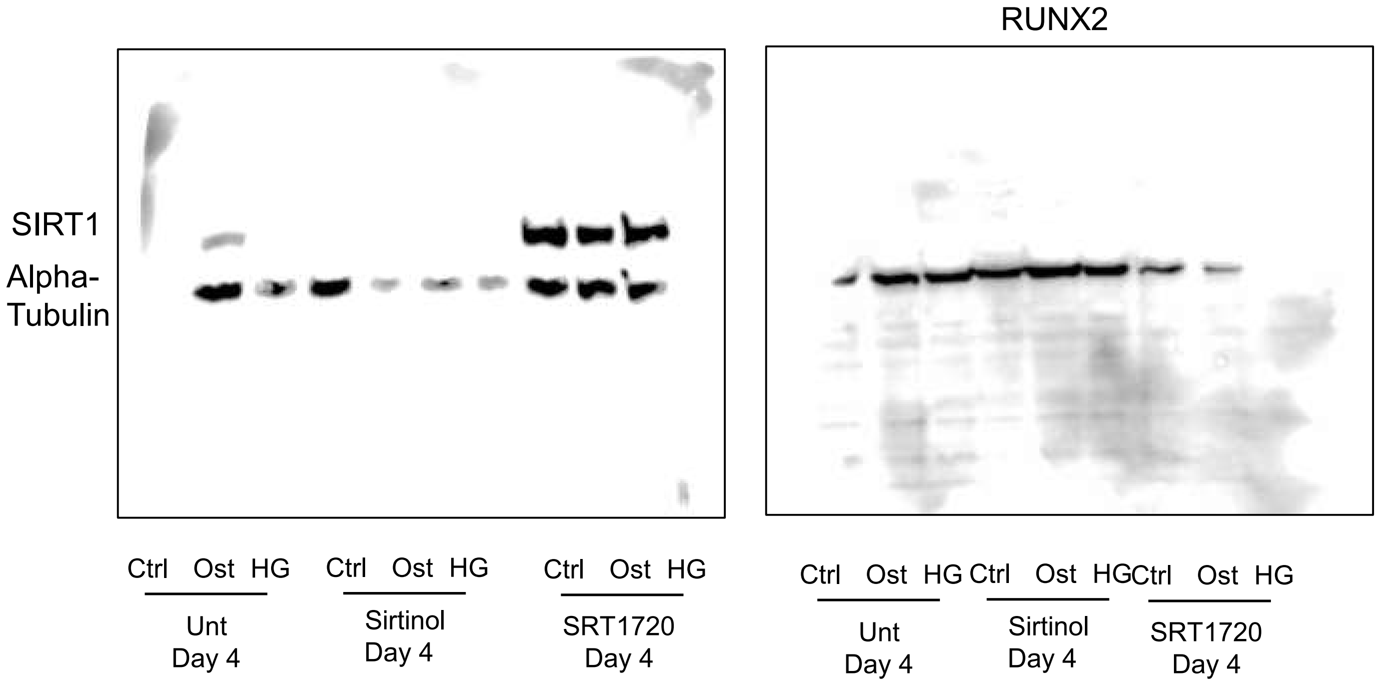

Supplementary Fig. S15.
Untreated & Sirtinol SIRT1, RUNX2 and Alpha-Tubulin Western blot.

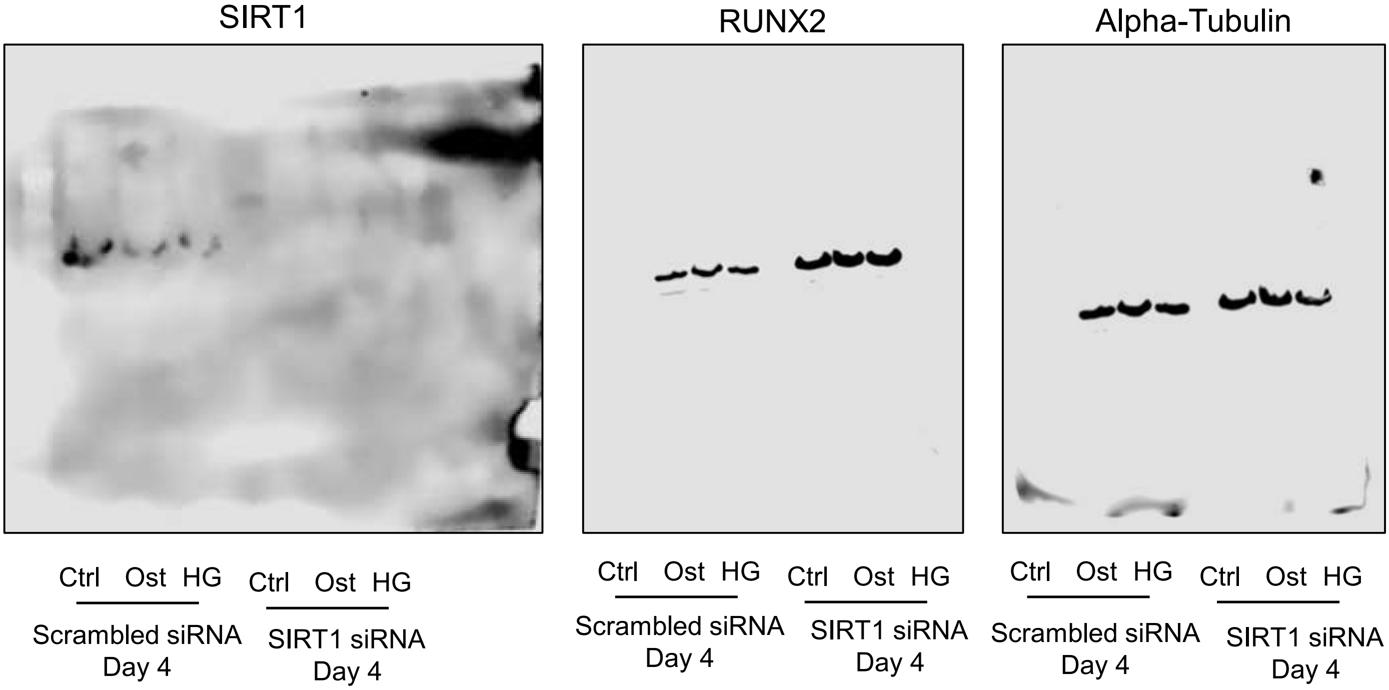

Supplementary Fig. S16.
Scrambled siRNA and SIRT1 siRNA SIRT,1RUNX2 and Alpha-Tubulin Western blot.
